# Supplementary material for: Efficient phylogenetic tree inference for massive taxonomic datasets: harnessing the power of a server to analyze 1 million taxa
Source: Gigascience. 2024 Aug 8;13:giae055. doi: 10.1093/gigascience/giae055 (PMC11308190; doi:10.1093/gigascience/giae055)
Supplement: giae055_GIGA-D-23-00320_Revision_1 [file giae055_giga-d-23-00320_revision_1.pdf]

GigaScience

Efficient phylogenetic tree inference for massive taxonomic datasets: harnessing the power of a server to analyze one million taxa

--Manuscript Draft--

Manuscript Number:	GIGA-D-23-00320R1
Full Title:	Efficient phylogenetic tree inference for massive taxonomic datasets: harnessing the power of a server to analyze one million taxa
Article Type:	Technical Note
Funding Information:	Ministerio de Ciencia e Innovación (PLEC2021-007662) — Dr Juan Carlos Pichel<br>Ministerio de Ciencia e Innovación (PID2022- 137061OB-C22) — Dr Juan Carlos Pichel<br>Xunta de Galicia (ED431G 2019/04) — Not applicable<br>Xunta de Galicia (ED431F 2020/08) — Dr Juan Carlos Pichel<br>Xunta de Galicia (ED431C 2022/16) — Not applicable<br>European Regional Development Fund — Not applicable
Abstract:	Background: Phylogenies play a crucial role in biological research. Unfortunately, the search for the optimal phylogenetic tree incurs significant computational costs, and most of the existing state-of-the-art tools cannot deal with extremely large datasets in reasonable times.<br>Results: In this work, we introduce the new VeryFastTree code (version 4.0), which is able to construct a tree on one server using single precision arithmetic from a massive one million alignment dataset in only 36 hours, which is 3x and 3.2x faster than its previous version and FastTree-2, respectively. This new version further boosts performance by parallelizing all tree traversal operations during the tree construction process, including subtree pruning and regrafting (SPR) moves. Additionally, it introduces significant new features such as support for new and compressed file formats, enhanced compatibility across a broader range of operating systems, and the integration of disk computing functionality. The latter feature is particularly advantageous for users without access to high-end servers, as it allows them to manage very large datasets, albeit with an increase in computing time.<br>Conclusions: Experimental results establish VeryFastTree as the fastest tool in the state-of-the-art for maximum-likelihood phylogeny estimation. It is publicly available at https://github.com/citiususc/veryfasttree. In addition, VeryFastTree is included as package in Bioconda, MacPorts and all Debian-based Linux distributions.
Corresponding Author:	Juan Carlos Pichel<br>Universidade de Santiago de Compostela<br>Santiago de Compostela, SPAIN
Corresponding Author Secondary Information:	
Corresponding Author's Institution:	Universidade de Santiago de Compostela
Corresponding Author's Secondary Institution:	
First Author:	César Piñeiro
First Author Secondary Information:	
Order of Authors:	César Piñeiro<br>Juan Carlos Pichel
Order of Authors Secondary Information:	
Response to Reviewers:	Efficient phylogenetic tree inference for massive taxonomic datasets: harnessing the

Powered by Editorial Manager® and ProduXion Manager® from Aries Systems Corporation

power of a server to analyze one million taxa
César Piñeiro and Juan C. Pichel

We would like to thank the reviewers and editors for their insightful comments and suggestions about the paper. All the changes in the revised manuscript are highlighted in red. Detailed responses to reviewers are given below.

Reviewer #1: This paper presents VeryFastTree version 4, an update of the VeryFastTree method published initially in 2020.
The new version features an advanced parallelization scheme that results in utilizing the maximum number of threads over nearly the full runtime of the program. Together with memory and other technical improvements, this makes VFT4 the fastest ML tree builder available, as the authors demonstrate in benchmarks. VFT4 also handles compressed file formats and features a disk computing functionality that stores data on the disk instead of RAM. For users with limited RAM machines, the latter feature enables them to compute trees, which would otherwise not be possible without upgrading RAM.

####################
####################

The paper is well written and I only have minor suggestions:

1) The abstract is very short and does not mention the new features build in VFT4. The features could be briefly mentioned (for those readers that don't read the full text).

****RESPONSE*****

Following the suggestion of the reviewer, we have extended the abstract information in the revised manuscript with the aim of including briefly some of the new features of VFT4 (page 1).

####################
####################

2) It would be helpful to add a figure that illustrates the new parallelization scheme. For example, the penalty parameter and parent / uncle swap is difficult to understand without seeing an illustration, especially because the penalty parameter is important for the new tree partitioning method.

****RESPONSE*****

We have significantly revised and expanded the section detailing the parallelization strategy of VFT4, spanning pages 2, 3, and 4 of the manuscript. These revisions include the incorporation of two new figures, one of which serves to illustrate the tree partitioning process and the role of the penalty value (Figure 1, page 2). A pseudocode explaining the tree partitioning algorithm was also included (Figure 2, page 3).

####################
####################

3) Why is level 3 and not level 4 the default level? Level 4 is faster and the authors mention that level 4 produces slightly higher accuracy. Is there a downside to level 4 parallelization?

****RESPONSE*****

Level 3 performs in parallel all the computations but SPRs, while Level 4 leverages the tree partitioning method to accelerate also SPR movements. However, the

Powered by Editorial Manager® and ProduXion Manager® from Aries Systems Corporation

parallelization of the SPR only pays off when the number of alignments is high. In other words, when the tree is large enough. For this reason, Level 3 is chosen by default as the best starting option for the users.

We have clarified this question in the revised manuscript (page 4).

###################
###################

4) VFT4 is non-deterministic, which is great. My understanding is that the repeated runs of VFT4 with the same parameters result in identical results. However, how can users tell VFT4 to introduce variation in the tree space search? Is there something like a seed parameter that initializes a random number generator? If so, the user could test if different seeds result in similar or identical trees.

****RESPONSE*****

The reviewer is right. There is an argument to set the seed for the random number generator: "-seed n". Its default value is 1253. Now this option is explained in the "Commands interface" section of the revised text (page 10), and it was specified that accuracies for all the tools were computed using the same seed (page 9). Since the behavior observed for VFT3, VFT4 and FT2 using different seeds is equivalent to the one detailed in Table 2, we only provide accuracy results for one seed.

___

Reviewer #2: The paper presents VFT4 - an upgrade version of the authors' own tool VFT3. The new updates as described in the paper include the application of SPR for exploring the tree space as well as the support of new file formats (nexus and compressed files). The authors mainly compare the performance of VFT4 with VFT3 and FT2. My main concern with this manuscript is that there is insufficient detail on the new features of VFT4, often times paragraphs read too vague to understand. This of course does not mean the work should not be published; on the contrary I believe improvements to phylogenetic inference are important but the paper requires a substantial revision to explain its functionality, the concepts involved as well as experiments to demonstrate/evaluate its accuracy.

Please find below a couple more specific comments:

###################
###################

* The manuscript does not include any information regarding the scoring function used for ranking phylogenies. Does the tree search aim to find the maximum likelihood estimates of the parameters and the MLE tree, or is the search based on parsimony (e.g. the number of changes in the tree) or a combination of both approaches? The authors should clarify this.

****RESPONSE*****

As it was explained in [1], VFT preserves the phases, methods and heuristics used by FT2 to estimate the phylogenetic tree. As a consequence, VFT (and FT2) uses a method based on maximum-likelihood (ML) to estimate phylogenetic trees. The maximum likelihood method seeks to find the tree topology and branch lengths that maximize the likelihood of the observed sequence data given the evolutionary model and parameters. This approach accounts for the probabilities of different evolutionary events (such as substitutions, insertions, and deletions) and attempts to find the most likely evolutionary history given the data and model assumptions.

Therefore, VFT does not use a parsimony-based approach. While parsimony is a valid method for phylogenetic inference, VFT emphasizes maximum likelihood estimation due to its efficiency and scalability for large datasets.

Powered by Editorial Manager® and ProduXion Manager® from Aries Systems Corporation

This information is included in the Introduction of the revised manuscript that enumerates some of the most important state-of-the-art tools for maximum-likelihood phylogeny estimation (page 1).

[1] Piñeiro C, Abuín JM, Pichel JC. VeryFastTree: speeding up the estimation of phylogenies for large alignments through parallelization and vectorization strategies. Bioinformatics 2020;36(17):4658–4659.

####################
####################

Also, based on some comments on "parent" and "uncle" nodes, it's unclear whether the program operates on rooted or unrooted trees. While recognizing that such information might have been described in prior publications, it's important for each manuscript to stand independently in this regard.

****RESPONSE*****

VFT uses rooted trees. Now this information was included in the revised text (page 2, "Tree partitioning" subsection).

####################
####################

* The manuscript needs a thorough discussion on how the correctness of results is assessed. Have simulation studies been conducted to evaluate the performance of the algorithm? Are the results compared against known phylogenies or validated against other phylogenetic software. If such comparisons are made, please specifying the software packages and datasets used for validation. To establish the reliability of VFT4, it would be advantageous to perform simulation studies using a set of datasets generated from real data. This approach would allow for the assessment of how frequently the program converges to the true phylogeny . Furthermore, conducting empirical analyses and comparing the results obtained from VFT4 with established software packages such as RAxML or IQ-TREE would instill confidence in the program's accuracy and robustness.

****RESPONSE*****

Correctness:

In the literature, several studies have examined the performance of leading ML tools, such as RaxML, IQ-TREE, and FT2, regarding accuracy and computational efficiency with large datasets. Liu et al. [1] compared RAxML and FT2 using simulated datasets for single genes containing 1000 or more sequences across various estimated alignments. They concluded that both methods exhibited similar topological accuracy. Lees et al. [2], in their exploration of ML heuristics through simulations, found RAxML and IQ-TREE to be comparable in accuracy, both outperforming FT2. Moreover, two studies [3,4] evaluated FT2 and RAxML on datasets containing fragmentary sequences. They observed that FT2 performed less accurately than RAxML when handling alignments with a high proportion of fragmentary sequences. Overall, these studies have shown that IQ-TREE and RAxML are both very good ML heuristics with respect to ML scores, while all of the studies have shown that FT2 is indeed very fast, but it is not very good at ML score optimization.

On the other hand, as we explained in our previous paper [5], VFT is a highly tuned implementation of FT2 that keeps the phases, methods and heuristics used by FT2 to estimate the phylogenetic tree. For this reason, it is only necessary to compare the topological accuracies with respect to FT2 (and our previous version of VFT), since the previous works performed a thorough comparison between RAxML, IQ-TREE and FastTree-2. This study was shown in the original paper in page 6, concluding that VFT4 produces trees with an accuracy level within the same range as FT2. We have used 5,000 sequences simulated protein alignments, which were also employed in the original FT2 paper for the same purpose. Therefore, the true phylogeny is available. All simulations are based on trees from profile alignments of biological sequences and

include variable rates across sites. The simulations are described in more detail in [6].

Now we have included the above information in the revised manuscript (page 8 - "Topological accuracy" subsection). FT2 and VFT versions are detailed in page 6. We have also added five new references to the bibliography. In addition, the dataset containing the 5,000 sequences simulated protein alignments used in the topological accuracy experiments is now included in the "Availability of supporting data and materials" section (page 11).

[1] Liu, K., Linder, C.R., Warnow, T. RAxML and FastTree: Comparing two methods for large-scale maximum likelihood phylogeny estimation. PLoS ONE 2011, 6, e27731.
[2] Lees, J.A., Kendall, M., Parkhill, J., Colijn, C., Bentley, S.D., Harris, S.R. Evaluation of phylogenetic reconstruction methods using bacterial whole genomes: A simulation based study. Wellcome Open Res. 2018, 3, 33.
[3] Smirnov, V., Warnow, T. Phylogeny estimation given sequence length heterogeneity. Syst. Biol. 2021, 70, 268–282. \\
[4] Sayyari, E., Whitfield, J.B., Mirarab, S. Fragmentary gene sequences negatively impact gene tree and species tree reconstruction. Molecular Biology and Evolution. 2017, 34, 3279–3291.
[5] Piñeiro C, Abuín JM, Pichel JC. VeryFastTree: speeding up the estimation of phylogenies for large alignments through parallelization and vectorization strategies. Bioinformatics 2020;36(17):4658–4659.
[6] Tatusov, R.L., Natale, D.A., Garkavtsev, I.V., Tatusova, T.A., Shankavaram U.T., et al. The COG database: new developments in phylogenetic classification of proteins from complete genomes. Nucleic Acids Res. 2001, 29: 22–8.

Running times:

As we mentioned, besides FT2 and VFT, there exist other state-of-the-art tools for ML tree estimation, notably RAxML and IQ-TREE, widely embraced by the scientific community. However, they are limited in their ability to process datasets containing a large number of sequences due to their extensive running times. To validate the observations made in previous works, we evaluated RAxML-NG v.1.2.0 and IQ-TREE v.2.1.3 using our Large dataset. Since RAxML-NG can be executed on a cluster, we conducted experiments with this tool using four computing nodes instead of just one server. We manually constrained the experiments to a maximum running time of one week. Both tools were unable to construct the tree within that time frame. It is worth noting that VFT4 can estimate the tree for the Large dataset in approximately 3 hours. This reiterates the unsuitability of RAxML and IQ-TREE for handling datasets with a substantial number of sequences. This information can be found in the revised manuscript (page 6).

####################
####################

* I find it hard to believe that single precision is beneficial for trees of that size (even for the Large dataset). Trees of such height will inevitably suffer from underflow when calculating conditional probabilities. How do you account for that? Even if some sort of numerical scaling is employed (e.g. adding a constant when reaching a threshold) I assume it is happening so often that it negates any benefit over using double precision (with scaling). Some discussion on this would be very useful.

****RESPONSE*****

Certainly, VFT implements numerical scaling to mitigate underflow, primarily through normalization techniques, adding constants or logarithmic values. Nonetheless, a minimum threshold is established to identify closely-related sequences, prompting user notification regarding calculation challenges and encouraging the adoption of double precision. Even if single precision is selected, many intermediate calculations such as vector reductions are performed with double precision to minimize errors, although final results influencing conditional probabilities are reverted to single precision.

This information is now included in the revised text (page 6).

Powered by Editorial Manager® and ProduXion Manager® from Aries Systems Corporation

####################
####################

* "New parallel regions"
The discussion on parallelization of tree traversals doesn't sound right. The overhead involved with traversing a tree structure, even with millions of nodes, is negligible compared to evaluating the scoring function (likelihood/parsimony/etc). My understanding is that a tree traversal involves generating an ordering for the tree nodes (e.g. postorder). It appears the authors may be referring to parallelizing the entire process of topology and evaluation, but this requires clarification. Moreover, the purpose of tree partitioning and the utilization of breadth-first traversal in this context lacks explanation. The text suggests that the tree is divided into disjoint graphs so that threads can work independently (although this "work" is not explained), yet it still happens that threads may need to operate outside of their partitioned graph, and a penalty parameter is used to avoid this.
Furthermore, it's unclear how topological changes (if that's what the authors describe) and branch lengths optimizations are executed in parallel on independent subtrees. Particularly, if the likelihood function is used for scoring, it is expected that changes necessitate re-evaluation of the likelihood by updating the conditional probabilities along the path to the root, assuming the remaining part of the tree is fixed (which I understand is not the case if you are working on different parts in parallel). This is not described in the manuscript.

****RESPONSE*****

Responding to several queries from the reviewer regarding the clarification and enhancement of the explanation of the parallelization process in VFT4, we have extensively revised and expanded the corresponding section, which now spans pages 2, 3, and 4 of the manuscript. These revisions include the introduction of two new figures (Figures 1 and 3), the inclusion of one pseudocode (Figure 2), and the addition of three new references [10-12] to aid in understanding.

####################
####################

* Finally, the section ends with the statement "On the other hand, in the parallel traverse approach, nodes at the same height level are computed simultaneously", which lacks clarity regarding what is computed and the methodology employed. The remaining part of the paragraph contains several undefined terms, such as "initial profiles" further exacerbating the confusion.

****RESPONSE*****

We have completely rewritten the text about the parallel breadth-first traversal (new subsection in page 4 of the revised manuscript): "Tree partitioning enables parallel processing of a single tree by assigning disjoint subtrees to each thread, allowing them to compute independently and without interference. However, when an operation has no dependencies and solely modifies the node being computed, creating subtrees is inefficient, as each node can be directly assigned to a thread. So, starting from the deepest nodes of the tree, each thread handles a portion of the nodes and computes them simultaneously, following a breadth-first traversal pattern, which involves visiting all nodes at a given depth level before moving on to the next level. Once finished with that level, the upper levels are distributed until the root of the tree is reached. This approach is specifically utilized in the following operations: computing initial profiles, recomputing profiles, recomputing ML profiles, computing tree length, and computing the likelihood for each site."

On the other hand, FT2 and VFT implements Neighbor-Joining by storing profiles for the internal nodes in the tree instead of storing a distance matrix with the aim of reducing the memory footprint. This information was detailed in the original FastTree papers [1,2]. Each profile includes a frequency vector for each position, and the weighted average of its children’s profiles. FT2 and VFT use these profiles to compute the distances between internal nodes in the tree and also the total distance from a node to all other nodes. Now we have clarified this in the paper (page 4) and we also

refer to the FT papers for details.

[1] Price MN, Dehal PS, Arkin AP. FastTree: computing large minimum evolution trees with profiles instead of a distance matrix. Molecular biology and evolution 2009;26(7):1641–1650.
[2] Price MN, Dehal PS, Arkin AP. FastTree 2 - Approximately Maximum-Likelihood Trees for Large Alignments. PLOS ONE 2010;5(3):1–10.

####################
####################

To enhance clarity, I advise to replace this section with a more intuitive explanation of the parallelization strategy. This revised section should delineate what aspects of the program were parallelized, the previous state of the program and the benefits accrued from parallelization. A figure illustrating the parallelization would greatly enhance the presentation of the manuscript. Technical intricacies can be relegated to supplementary material for readers seeking detailed understanding.

****RESPONSE*****

As previously mentioned, in response to the reviewer's suggestion, we have undertaken substantial revisions and expansions to the section elucidating the parallelization strategy of VFT4 (pages 2, 3 and 4 of the revised manuscript). This includes a detailed explanation of the tree partitioning algorithm, the mapping of subtrees to threads, the proposed stopping criterion aimed at reducing the search space when seeking the best partitioning, and the parallel breadth-first traversal.

####################
####################

* Non-deterministic deprecation

This section lacks informative content and delves excessively into details without contextualization. To improve clarity, replace the sentence 'certain sections of the code perform synchronized simultaneous modifications using mutex (a mechanism for controlling access to shared resources)' with 'critical regions', a standard term in the context of parallelizations. If the reader is not familiar with such a term, they will certainly not understand concepts like 'mutex' either. Additionally, the paragraph discusses some critical region, which had a non-deterministic behaviour, became deterministic, then it evolved back to non-deterministic again, and now, in VFT4, it's changed again to determinstic, without describing its function, the nature of deterministic and non-deterministic actions within it, or the implications for speed and memory footprint. I don't think this paragraph provides any meaningful information to the reader (at least not to me), and perhaps consider either clarifying or deleting it.

****RESPONSE*****

After careful consideration, we recognize that the paragraph discussing determinism may not provide meaningful information to readers, especially without sufficient context or explanation of its implications. Given its technical nature, we have opted to remove this paragraph from the manuscript.

####################
####################

* Some principles appear ot be misused, particularly regarding memory categorization. On page 3, the authors classify program memory (RAM) into static, dynamic and computation categories. However, the term "static memory" traditionally refers to memory that is allocated at compile time, and which remains unchanged throughout program execution. It appears that all three memory types mentioned by the authors are part of dynamic memory, allocated during the program execution. The description suggests that some dynamically allocated memory is transferred to disk when not actively utilized, a process which is akin to swapping managed by the operating system. The rationale behind implementing manual memory swapping within the

program raises questions, especially considering that modern operating systems already employ sophisticated swapping mechanisms. Leveraging OS-managed swapping could obviate the need for custom memory management within the program, and thus the obvious question is why not just let the OS do the swapping when necessary?
Moreover, the manuscript lacks clarity regarding the specific contents of memory being swapped to disk. While the authors mention static and dynamic memory is subjected to swapping (in my terminology, any kind of memory allocated by the program at run-time), it remains unclear whether that encompasses things like intermediate likelihood/parsimony results at each node, which should be accessible by each thread (i.e. shared memory) and form the main bulk of memory scheme. Finally, I would imagine that the part of memory described as "computation" should be tiny compared to intermediate likelihood scores. Or do you refer to local NUMA memory as "computation"? In any case, describing the precise memory contents slated for swapping would be greatly appreciated by other software developers in the field.

****RESPONSE*****

The reviewer is right, as all memory types discussed in the text pertain to dynamically allocated memory. To alleviate any confusion, we have opted to rename these memory types and explicitly specify that we are exclusively referring to types of dynamic memory. First, fixed memory (previously static) refers to memory allocated at the start of the program, once the properties of the input dataset (such as the number of sequences, length, etc.) are established. For instance, fixed memory is used to store sequences and weights in each profile. Variable memory (previously dynamic) is the memory used to store the frequency vector in each profile (conditional probabilities) whose size changes dynamically and cannot be precisely predicted. Finally, computation memory refers to the requirements of temporary memory that are released after producing an intermediate result.

On the other hand, using swap space as an alternative when a process exceeds RAM capacity has important limitations and drawbacks. First, the swap partition has a fixed size, potentially leading to out-of-memory errors if exceeded. This is especially relevant when dealing with very large datasets, as is our case. Therefore, if the dataset exceeds the size of the swap, only an administrator would have the authority to expand the swap partition to prevent the job from being aborted. In addition, since all programs share the same swap space, excessive swapping by one program can adversely affect others, leading to contention for disk I/O resources and reduced responsiveness. In contrast, the mechanism used by VFT4 in "disk computing" is based on memory-mapped files, which offers better I/O performance due to the optimized kernel-level caching mechanisms and efficient block-based access. Note that when using memory mapping any available disk can be used to store data, avoiding the swap partition size limitation. Additionally, memory-mapped files are also controlled by the operating system, similar to swap space, and are not manually managed. This ensures efficient handling of data movement between memory and the disk, with the operating system making decisions based on system resources and requirements.

Finally, computation memory, even for huge datasets, remains significantly smaller in comparison to fixed and variable memories.

We have included and extended the above information in the "Disk computing" section (pages 4-5).

###################
###################

* Performance evaluation: please explain what the -spr 4 -gamma -gtr switches mean. I assume a GTR+Gamma model is used with a discretized gamma approximation for rate heterogeneity using 4 categories, but this should be stated explicitly. Also, explain what the -spr 4 switch does.

****RESPONSE*****

-> -nt option: Nucleotides alignment used as input.

Powered by Editorial Manager® and ProduXion Manager® from Aries Systems Corporation

-> -spr option: Set the number of rounds of subtree-prune-regraft (SPR) moves (default value is 2).
-> -gtr option: The generalized time-reversible (GTR) model of nucleotide substitution is used.
-> -gamma option: VFT (and FT2) will report the likelihood under the discrete gamma model with 20 rate categories ("Gamma20"). More precisely, after optimizing the tree with a fixed rate for each site (the CAT model), VFT (and FT2) will rescale the tree to optimize the Gamma20 likelihood. That is explained and justified in the original paper of FT2 [1].

Now this information was included in the revised manuscript (pages 5-6).

[1] Price MN, Dehal PS, Arkin AP. FastTree 2 - Approximately Maximum-Likelihood Trees for Large Alignments. PLOS ONE 2010;5(3):1–10.

####################
####################

* Figure 1 and Figure 2 do not seem to match the text. I think the third cluster of bars (Ultra-large dataset) should be swapped between the figures?

****RESPONSE*****

There was an error in the Y axis (Time) in Figure 2 (double precision running times). Now this was solved in the revised text (now Figure 5, page 7). In addition, we have included the following explanation about the lower time obtained with the large dataset and double precision compared to the time observed for single precision (page 6): "Note that VFT4 builds the tree faster when using double precision than considering single precision (2.8h versus 3.5h). This is caused by the good behavior of the vectorization strategies used by VFT4 when considering protein alignments."

####################
####################

* Figure 3. Assuming VFT4 involves ML estimation using GTR-G4 and minimal site compression due to the amount of sequences, the memory requirement for the Ultra-Large dataset would be roughly ~ 1.4 TB [inner nodes * sites * states * rates * sizeof(float) = (989109-2)*21946*4*4*4] and that's accounting for only the conditional probabilities at inner nodes. Similarly, figs 4ab shows memory usage for the "Large" dataset using double precision. Based on my calculations the expected memory required for storing the conditional probabilities should be (274401-2)*1287*20*4*8 ~= 230 GB (assuming no site compression), yet the plotted max memory usage at a time is at most ~ 80 GB with 75\% of time being at ~ 10GB. This is a huge discrepancy and should be explained/discussed whether VFT4 is doing something very smart or if there is a huge amount of gappy or identical sites affecting memory use.

****RESPONSE*****

As we pointed out previously, VFT4, like FT2, use sequence profiles of internal nodes of the tree to implement Neighbor-Joining instead of storing a distance matrix. Typically, as it was stated in [1], a distance matrix for an alignment with 'N' sequences, 'L' sites, and 'a' different characters in the alphabet, requires $O(N^2)$ space. However, the upper bound of memory space required by VFT4 and FT2 is $O(NLa)$. Each profile comprises a frequency vector for each position, and the weighted average of its children’s profiles.

If we consider the Ultra-Large dataset, whose required memory is 272 GB, its corresponding parameters are: N = 989,109, L = 21,946, and a = 4 (nucleotides). As a consequence, taking into account that the resulting tree contains 2N nodes and single precision uses 4 bytes, the memory required by the profiles is the sum of the weights and frequencies for each node in the tree:

-- Weights require exactly 2N*L*4 bytes = (2 * 989,109) * 21,946 * 4 bytes = 173.65 GB.

Powered by Editorial Manager® and ProduXion Manager® from Aries Systems Corporation

	-- The memory of frequencies can be estimated as: 2N*a*g*4 bytes, being 'g' dependent on the number of gaps in the sequence. Since 'g' is not a constant value and changes in each node as the topology of the tree evolves, an exact value for the memory space used cannot be provided. In any case, most of the difference between the total memory used by VFT4 (272 GB) and the memory required only by the weights of the profiles (173.65 GB) is due to the frequencies.<br><br>Now we have included the above information in the revised manuscript (page 7).<br><br>[1] Price MN, Dehal PS, Arkin AP. FastTree: computing large minimum evolution trees with profiles instead of a distance matrix. Molecular biology and evolution 2009;26(7):1641–1650.<br><br>###################<br>###################<br><br>- Could you provide more insight into memory usage and program flow, particularly in relation to figures 4a and 4b? The plots indicate that the program typically maintains a modest memory footprint, averaging around 15-20 GB for the majority of its runtime. However, there is a notable spike in memory usage to approximately 80 GB at certain points. What is the reason behind this? How do you store intermediate results (conditional probabilities)?<br><br>****RESPONSE*****<br><br>The spike in the memory consumption is caused by the computation and storage of the profiles at each node of the tree. This information is now included in the revised text (page 8). The conditional probabilities, as we mentioned previously, were calculated using a profile (a C struct in the implementation). Each profile includes a frequency vector for each position, and the weighted average of its children's profiles.<br><br>###################<br>###################<br><br>- The manuscript introduces several concepts such as level 3 and level 4 parallelization, as well as terms like "partitioning tendency window" and the utilization of a "tree partition algorithm" to accelerate spr movements. However, these concepts are mentioned without adequate explanation. It would greatly enhance the clarity of the manuscript to provide detailed explanations of these concepts, preferably accompanied by a figure for visual aid.<br><br>****RESPONSE*****<br><br>All these concepts have been clearly explained in the new revised "Parallelization strategy" section (pages 2, 3 and 4). As suggested, we have incorporated two new figures and a pseudocode detailing the tree partitioning algorithm to enhance clarity. Furthermore, we have provided clarification on the distinctions between different thread levels in the "New thread levels" section on page 4.
**Additional Information:**	
**Question**	**Response**
Are you submitting this manuscript to a special series or article collection?	No
**Experimental design and statistics**<br><br>Full details of the experimental design and statistical methods used should be given in the Methods section, as detailed in our Minimum Standards Reporting Checklist.	Yes

Powered by Editorial Manager® and ProduXion Manager® from Aries Systems Corporation

Information essential to interpreting the data presented should be made available in the figure legends.<br><br>Have you included all the information requested in your manuscript?	
**Resources**<br><br>A description of all resources used, including antibodies, cell lines, animals and software tools, with enough information to allow them to be uniquely identified, should be included in the Methods section. Authors are strongly encouraged to cite Research Resource Identifiers (RRIDs) for antibodies, model organisms and tools, where possible.<br><br>Have you included the information requested as detailed in our Minimum Standards Reporting Checklist?	Yes
**Availability of data and materials**<br><br>All datasets and code on which the conclusions of the paper rely must be either included in your submission or deposited in publicly available repositories (where available and ethically appropriate), referencing such data using a unique identifier in the references and in the “Availability of Data and Materials” section of your manuscript.<br><br>Have you have met the above requirement as detailed in our Minimum Standards Reporting Checklist?	Yes

Manuscript

Click here to access/download;Manuscript;VeryFastTree4___GigaScience_

OXFORD

(GIGA)^n SCIENCE

GigaScience, 2017, 1–11

doi: xx.xxxx/xxxx
Manuscript in Preparation
Paper

PAPER

Efficient phylogenetic tree inference for massive taxonomic datasets: harnessing the power of a server to analyze one million taxa

César Piñeiro[1,*,†] and Juan C. Pichel[2,*]

[1]Information Retrieval Lab, CITIC, Universidade da Coruña, 15008 A Coruña, Spain and [2]CiTIUS, Universidade de Santiago de Compostela, 15782 Santiago de Compostela, Spain

[*]cesaralfredo.pineiro@usc.es; juancarlos.pichel@usc.es
[†]Corresponding author

Abstract

Background: Phylogenies play a crucial role in biological research. Unfortunately, the search for the optimal phylogenetic tree incurs significant computational costs, and most of the existing state-of-the-art tools cannot deal with extremely large datasets in reasonable times.
Results: In this work, we introduce the new VeryFastTree code (version 4.0), which is able to construct a tree on one server using single precision arithmetic from a massive one million alignment dataset in only 36 hours, which is 3× and 3.2× faster than its previous version and FastTree-2, respectively. This new version further boosts performance by parallelizing all tree traversal operations during the tree construction process, including subtree pruning and regrafting (SPR) moves. Additionally, it introduces significant new features such as support for new and compressed file formats, enhanced compatibility across a broader range of operating systems, and the integration of disk computing functionality. The latter feature is particularly advantageous for users without access to high-end servers, as it allows them to manage very large datasets, albeit with an increase in computing time.
Conclusions: Experimental results establish VeryFastTree as the fastest tool in the state-of-the-art for maximum-likelihood phylogeny estimation. It is publicly available at https://github.com/citiususc/veryfasttree. In addition, VeryFastTree is included as package in Bioconda, MacPorts and all Debian-based Linux distributions.

Key words: Phylogenetics; Very large datasets; Performance; Parallelism;

Introduction

Inferring evolutionary relationships or phylogenies is a formidable challenge in computational biology. The growth of datasets from next-generation sequencing has made large-scale phylogeny estimation crucial. However, the computational complexity of inferring phylogenies and performing multiple sequence alignment (MSA) presents a significant obstacle. Established methods like maximum-parsimony (MP), maximum-likelihood (ML), and Bayesian approaches are computationally intensive due to the NP-hard optimization problems they tackle [1]. As the number of taxa increases, these methods face a common hurdle: an exponential increase in the number of possible trees to explore.

The leading heuristics for ML tree estimation, such as RAxML [2] and IQ-TREE [3], employ diverse strategies to search for the tree that maximizes the likelihood score. Although they have made considerable performance improvements to handle larger datasets, RAxML, for example, was unable to reach convergence on a 10,000-sequence dataset even after a week [4]. Note that these tools are primarily optimized for datasets with a limited number of sequences but a significant number of sites (i.e., phylogenomics). Therefore, when working with datasets comprising a large number of sequences, users must opt for tools such as FastTree-2 [5] and VeryFastTree [6], which are very fast heuristics but they do not make very substantial attempts to optimize the likelihood score, or they may explore divide-and-conquer strategies [7, 8, 9]. In

Compiled on: April 17, 2024.
Draft manuscript prepared by the author.

particular, our tool VeryFastTree was a big step forward in terms of performance, building a tree on a standard server from a large 330k alignment, 3.5× faster than FastTree-2. However, there was still room for improving its speed, scalability, memory consumption and also to add new functionalities.

In this work, we introduce the latest VeryFastTree code, version 4.0, showcasing its potential advantages and new features compared to both its previous version and FastTree-2. The earlier iteration of VeryFastTree achieved high performance by parallelizing the most time-consuming phase of constructing the tree, specifically the Nearest-Neighbor Interchanges (NNIs), in comparison to FastTree-2. However, the new VeryFastTree further enhances performance by parallelizing all tree traversal operations, including, among others, the Subtree Pruning and Regrafting (SPR) operations, which are especially relevant in terms of computing time when dealing with massive datasets. After a thorough experimental evaluation, the new version proves to be several times faster than the previous one and FastTree-2 on a variety of large datasets. At the same time, VeryFastTree-4 incorporates significant new features, including support for new and compressed file formats, improved compatibility with a wider range of operating systems, and the addition of *disk computing* functionality. The latter is especially valuable for users who do not have access to high-end servers, as it allows them to process massive datasets, albeit with an increase in computing time.

New features and optimizations

VeryFastTree-4 (VFT4) is a big step forward with respect to our first version introduced in [6], from now on VFT3, designed to further accelerate the inference of phylogenies for massive alignments. Building upon the strengths of its predecessor, VFT4 introduces a host of innovative features and optimizations aimed at achieving even greater speed and efficiency. While the core principles used by VFT4 remain consistent with its previous versions, significant enhancements have been made to push the boundaries of speed. Below are some of the most noticeable improvements, optimizations and features incorporated into VFT4:

Parallelization strategy

VFT3 achieved high performance by parallelizing the most time-consuming phase in the construction of the tree with respect to FastTree-2 (FT2) [5], the NNIs. Building upon this progress, VFT4 continues this approach by parallelizing all operations involving tree traversal, including the SPR moves, further enhancing performance. These computations are parallelized using two strategies: *tree partitioning*, which divides the tree into multiple non-overlapping subtrees, and parallel traverse, a *parallel breadth-first traversal*.

Tree partitioning

Once an initial phylogenetic tree is constructed or passed as input argument, it is necessary to split the tree into disjoint subtrees in order to perform computations in parallel using different threads. Note that VFT4 uses rooted trees. The tree partitioning method is an algorithm that, given an initial tree, prunes nodes from the root to generate several independent subtrees, which are then assigned to different threads to work with them independently. Once all threads have finished their work, those nodes that were removed in the pruning process are subsequently processed using just one thread. In this way, all nodes in the tree are updated. In the ideal scenario, the computation of a node does not have dependencies on other nodes or these dependencies are contained within the subtree assigned to the same thread. However, this is not the case frequently, so dependencies must be controlled to prevent computation errors. For instance, NNIs and SPR operations perform topology-modifying actions on the tree. They require tree partitioning to ensure that node exchanges performed by different threads do not overlap. Tree partitioning must ensure that these operations can be executed in parallel within separate subtrees, allowing them to work independently without interfering with each other.

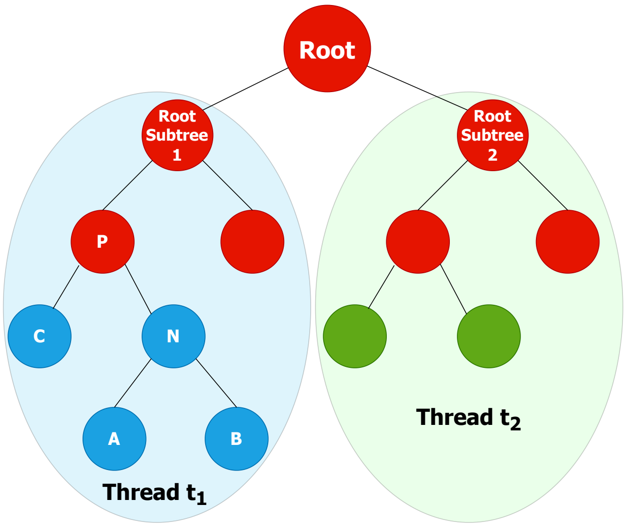

Figure 1. Example of tree partitioning and data dependencies with a penalty of two. The graph represents only internal nodes (no leaves). Blue and green nodes are processed in parallel by threads t_1 and t_2, respectively. Red nodes (labeled as *not-assigned*) are processed sequentially after processing all subtrees.

Before getting into the details, it is necessary to understand how the tree is traversed and how data dependencies between nodes are taken into account. We will use the tree of Figure 1 as example. Each node in the tree is visited before its parents, which means that a depth-first post-order traversal is used [5]. Let's assume that the tree is split into two subtrees assigned to threads t_1 and t_2, respectively. Since the root node of the tree is not assigned to a thread, it will be labeled as *not-assigned*. Blue and green nodes are processed in parallel by threads t_1 and t_2, while red nodes, which have dependencies on other threads, are processed sequentially after all subtrees have been processed. This ensures that all nodes in the tree are updated effectively. The dependencies of a node are determined by its relationships with its parents and siblings. If these are not located within the same subtree, the node cannot be computed. This dependency can be quantified as the distance between a node and the farthest one required for computation. In this way, we introduce the concept of *penalty* value, which will be defined as the number of levels from the subtree's root node that are excluded to ensure that all nodes have their dependencies satisfied within the subtree. In the example of Figure 1, the penalty is two, so P cannot be computed since its grandparent is the root, which lies outside the subtree. On the other hand, C, N, and all their children have their grandparents within the tree, allowing them to be computed without conflicts.

NNIs and SPR operations are parallelized using the tree partitioning strategy. In particular, NNIs perform a node exchange with either its parent or its uncle, followed by a recalculation of the weights of the involved nodes and their respective parents. This process incurs a penalty of two, as the parent or uncle will be one level above the exchanged node, while the new parent will be located at the next level. In the case of SPRs, the penalty is dynamic and constrained by the parameter `maxSPRLength`, which limits the maximum distance a node can move during the regrafting process. Other operations such as computing SH-like supports, updating all branch lengths, and optimizing all branch lengths will also make use of tree partitioning.

VFT4 implements an advanced partitioning algorithm when

compared to its predecessor, VFT3. This upgraded algorithm significantly improves both speed and adaptability across various scenarios. Unlike the previous version, which limited partitioning to NNIs, the new algorithm introduces a more versatile approach, allowing its application to other operations, such as those previously mentioned. This new algorithm considers the minimization of the penalty parameter as an evaluation criterion. Subsequently, the resulting subtrees generated by this process, also referred to as *solutions*, are assigned to threads with the aim of achieving a good load balance.

Partitioning method. Our partitioning method is guided by an objective function, whose goal is two-fold. First, it should balance the workload assigned to each thread. That is, the number of internal nodes to be processed by each thread. To model the workload we define the *weight* of a node as the number of internal nodes beneath it plus itself. This way, the *workload* associated to process a subtree is the weight of its root node. The second goal of the objective function should be to reduce to the minimum the amount of sequential work, which corresponds to processing the *not-assigned* nodes. Note that according to the Amdahl's law [10], a small percentage of sequential work harms noticeably the performance and scalability of a parallel application.

Let T be the input tree to be partitioned, which only contains internal nodes. A partition $P(T)$ is a set of disjoint binary subtrees s_i included in T. We must highlight that not all nodes in the tree should be included in a partition. Let $|P(T)|$ denote the number of subtrees in the partition and n the number of threads. Each subtree $s_i \in P(T)$ is assigned to a particular thread in such a way that $P_j(T)$ contains all subtrees in the partition $P(T)$ assigned to thread j. This way, we can calculate the workload of the subtrees assigned to a particular thread j as:

$$workload(P_j(T)) = \{\sum workload(s_i) \mid s_i \in P_j(T)\} \tag{1}$$

We evaluate a partition $P(T)$ using the following objective function:

$$\frac{sequential\_workload}{\max\{workload(P_j(T)) \mid 1 \leq j \leq n\}} \tag{2}$$

Note that *sequential_workload* refers to the workload of T when is processed sequentially. The goal of our partitioning method will be to find a partition $P(T)$ that maximize the value of the objective function. The pseudocode of the algorithm to find the best tree partitioning is detailed in Figure 2. The objective function aligns with the concept of *Speedup*, defined as the ratio of the execution time of a task when using multiple threads compared to the execution time when using a single thread. In this way, a value of 1 indicates equivalent speed to sequential processing, while a value of n signifies optimal workload distribution among n threads.

The tree partitioning process in VFT4 includes an initialization phase where the weight values for each node in the tree are computed. As previously mentioned, these weights represent the number of descendant nodes for each node, adjusted according to the penalty value. Then, the initial solution is constructed using the direct child nodes of the tree's root. In other words, the initial solution has as many subtrees as the root node has children. Nodes in the solution are always sorted by weight for efficiency. Next, an iteration process begins. During each iteration, the current solution is evaluated using Equation 2. To achieve this, it is necessary to assign subtrees to the different threads using a heuristic method, which will be explained later. Once the solution is evaluated, a new one is created in such a way that the subtree root node with the highest weight is replaced by its child nodes. The evaluation results of the last W iterations are stored to use them as stopping criterion. We refer to W as the *tendency window* *size*. Note that the algorithm continues as long as the current solution has nodes to split, there are threads without at least one subtree assigned, or the stopping criterion is not reached.

```
function TreePartition
Input  :tree, threads_num, penalty, tendency_size
Output :best_solution

// Initialization: initial solution and weights computation
weights ← ComputeWeights (tree, penalty)
solution ← Children (RootNode (tree))
best_solution ← solution
solution_speedup ← Evaluate (solution)
best_solution_speedup ← solution_speedup
tendency ← []
// Iterations to find the best solution
while Len (solution) > 0 ∧ (Len (solution) < threads_num ∨
 !StoppingCriterion (tendency, tendency_size)) do
    node ← GetAndRemoveNodeWithMaxWeight (solution)
    if node has children then
        Insert (Children (node)) into solution
    solution_speedup ← Evaluate (solution)
    Insert (solution_speedup) into tendency
    if solution_speedup > best_solution_speedup then
        best_solution ← solution
        best_solution_speedup ← solution_speedup
return best_solution

function StoppingCriterion
Input  :tendency, tendency_size
Output :must_stop
if Len(tendency) = tendency_size then
    balance ← 0
    for i ← 1 to tendency_size do
        for j ← i to tendency_size do
            if tendency_i <= tendency_j then
                balance ← balance + 1
            else
                balance ← balance - 1
    RemoveFirst (tendency)
    return balance < 0
return false

function ComputeWeights
Output :Weight of each node in the tree taking into account the penalty

function Children
Output :Childrens of the node

function GetAndRemoveNodeWithMaxWeight
Output :Gets and removes the node with the highest weight

function Evaluate
Output :Quality of the solution calculated considering Equation 2
```

Figure 2. Pseudocode of the method to find the best tree partitioning.

Assignment of subtrees to threads. The evaluation of the quality of a solution requires mapping the subtrees of a partition to the considered threads in such a way that the workload is balanced among them. This is a challenge itself and resembles the *k-partitioning* problem [11]. The k-partitioning problem is defined as follows: given a set of items $\{I_1, I_2, ..., I_n\}$ where item I_j is of weight $w_j > 0$, find a partition $S_1, S_2, ..., S_m$ of this set with $|S_i| = k$ such that the maximum weight of all subsets S_i is minimal. Therefore, considering our case, items are subtrees and k is the number of threads. The k-partitioning problem is NP-hard, so algorithms/heuristics to approximate the solution should be used.

Since our procedure requires solving the k-partitioning problem every time a new partition is evaluated, we have opted for a simple greedy approximation based on the first-fit decreasing algorithm whose computational cost is low. The method starts sorting the subtrees in descending order according to their weight. Then the k first subtrees are distributed among the threads. The remaining subtrees are iterated and assigned to the thread that currently has the minimum workload assigned. That is, the lowest

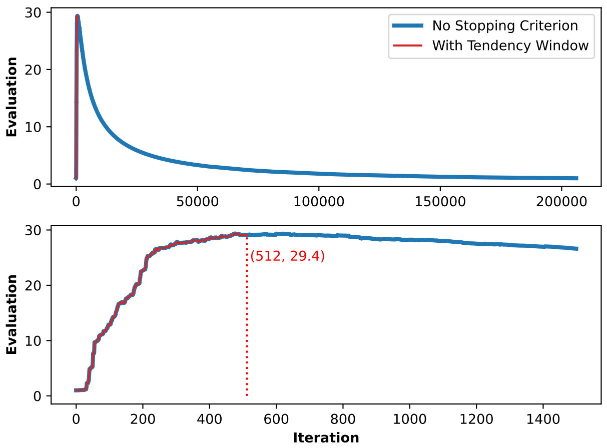

Figure 3. Comparison of iterations required for achieving near-optimal tree partitioning using our method versus evaluating all possible tree partitionings without a stopping criterion. The bottom figure provides a zoomed-in view of the top one. Experiments were conducted with 30 threads and a dataset consisting of 274K unique sequences.

value of $workload(P_j(T))$.

Stopping criterion. The stopping criterion in the tree partitioning process determines when the algorithm should terminate its execution. This criterion is required because completing all iterations, until no more nodes are left to split, can sometimes result in a higher computational cost than working on the tree without partitions. Therefore, the stopping criterion ensures that the algorithm halts its execution when a near-optimal solution has been found. To verify if the stopping criterion is met, we assess the quality of the last W partitions using Equation 2 to determine whether the overall trend in recent iterations indicates an improvement or deterioration in the solutions (see the `StoppingCriterion` function in Figure 2). In order to achieve this, a counter compares each pair of elements within the tendency list, incrementing it if the earlier element is less than or equal to the later element, and decrementing it otherwise. Consequently, the function returns true if the balance is negative, indicating a worsening trend, and false otherwise, signaling that the stopping criterion has not yet been met due to improving solutions. By default, W is set to 50, but users can modify this value through the interface parameter (see the Commands Interface section for details).

To demonstrate the advantages of our proposal, Figure 3 illustrates the comparison between the number of iterations required by our method to achieve a near-optimal tree partitioning and the scenario where all possible tree partitionings are evaluated without any stopping criterion. These results were obtained using 30 threads and the *Large* dataset that contains 274K unique sequences (refer to Table 1 for dataset details). Since 30 threads were considered, according to Equation 2, the ideal (maximum) quality value of a partitioning is 30. Our method only requires 512 iterations to reach a value of 29.4, while without stopping criterion is necessary to evaluate more than 200K partitionings (iterations).

Parallel breadth-first traversal

Tree partitioning enables parallel processing of a single tree by assigning disjoint subtrees to each thread, allowing them to compute independently and without interference. However, when an operation has no dependencies and solely modifies the node being computed, creating subtrees is inefficient, as each node can be directly assigned to a thread. So, starting from the deepest nodes of the tree, each thread handles a portion of the nodes and computes them simultaneously, following a breadth-first traversal pattern, which involves visiting all nodes at a given depth level before moving on to the next level. Once finished with that level, the upper levels are distributed until the root of the tree is reached. This approach is specifically utilized in the following operations: computing initial profiles, recomputing profiles, recomputing ML profiles, computing tree length, and computing the likelihood for each site. It is worth noting that FT2 and VFT4 store profiles for the internal nodes in the tree instead of storing a distance matrix, which requires far less memory. Each profile includes a frequency vector for each position, and the weighted average of its children's profiles. These profiles are used to compute the distances between internal nodes in the tree and also the total distance from a node to all other nodes (see [5, 12] for a detailed description).

New Thread Levels

To provide users control over parallelism and adaptability to various usage scenarios, VFT4 introduces five distinct thread levels. These levels, including the original levels from VFT3 (now referred to as level 0, 1 and 2), allow users to finely adjust the degree of parallelization based on their specific requirements.

At level 0, VFT4 employs the same parallelization strategy as FT2, but with the addition of new parallel blocks to enhance performance. Level 1 introduces parallel blocks that require additional memory for computations, enabling more efficient processing. Level 2 utilizes the tree partitioning method to accelerate ML NNIs (Maximum Likelihood Nearest Neighbor Interchanges) rounds. Level 3 performs in parallel all the computations but SPRs (Subtree Pruning and Regrafting). Lastly, level 4 leverages the tree partitioning method to expedite also SPR steps, but it only pays off with datasets containing a high number of alignments. For this reason, level 3 is the default option.

Each level in VFT4 is incremental with respect to the previous ones. However, it is important to note that computation at level 2 and above follows a different tree traverse order, which may result in different trees with respect to the sequential execution. Nevertheless, these results remain strictly correct. By incorporating this multi-level approach, VFT4 offers users the flexibility to optimize their parallelization strategy according to factors such as dataset size, performance needs, and desired trade-offs.

Disk Computing

As previously mentioned, phylogenetic tree inference requires a significant amount of memory, which can become problematic when dealing with numerous sequences and limited resources. For example, when considering a low-end server with reduced available RAM memory. To tackle this challenge, VFT4 introduces *disk computing*, a technique that utilizes the hard drive to supplement the memory requirements. While disk computing aids in handling large datasets, it does impact performance due to the slower access speed of hard disks compared to RAM. However, the advantages of being able to process larger datasets outweigh the performance trade-off.

We classified the dynamically allocated memory used in the tree construction process into three types: fixed memory, variable memory, and computation memory. In particular:

- *Fixed memory* is allocated at the beginning of the program execution once the properties of the input dataset (such as the number of sequences, length, etc.) are established, and remains reserved until the program's completion. For instance, fixed memory is used to store sequences and weights in each profile.
- *Variable memory*, on the other hand, is allocated to store values during specific stages of the computation. For instance, it is the memory used to store the frequency vector (conditional probabilities) whose size changes dynamically and cannot be precisely predicted. Note that due to significant variations in data sizes,

over-allocation of variable memory is often impractical.

- *Computation memory* refers to private memory required by threads to perform their tasks. This includes stack memory, which handles function calls, local variables, and temporary data while the program is running. Additionally, computation memory includes some temporary variables, which store intermediate results and facilitate complex calculations, as well as data structures, which organize and manage data for efficient processing. For example, computation memory is used to temporarily store the new computed conditional probabilities for possible exchanges within an NNI iteration. It is important to emphasize that once the temporary values are utilized, they are subsequently released.

While fixed and variable memory can be offloaded to the disk, computation memory must be always maintained in RAM. This is because the performance of the computation process is critical. In addition, even for huge datasets, computation memory remains significantly smaller in comparison to fixed and variable memories.

On the other hand, using swap space as an alternative when a process exceeds RAM capacity has important limitations and drawbacks. First, the swap partition has a fixed size, potentially leading to out-of-memory errors if exceeded. This is especially relevant when dealing with very large datasets, as is our case. Therefore, if the dataset exceeds the size of the swap, only an administrator would have the authority to expand the swap partition to prevent the job from being aborted. In addition, since all programs/applications share the same swap space, excessive swapping by one program can adversely affect others, leading to contention for disk I/O resources and reduced responsiveness. In contrast, the mechanism used by VFT4 in disk computing is based on memory-mapped files, which offers better I/O performance due to the optimized kernel-level caching mechanisms and efficient block-based access. Note that when using memory mapping any available disk can be used to store data, avoiding the swap partition size limitation. Additionally, memory-mapped files are also controlled by the operating system, similar to swap space, and are not manually managed. This ensures efficient handling of data movement between memory and the disk, with the operating system making decisions based on system resources and requirements.

VFT4 introduces the use of disk computing with two parameters:

- `-disk-computing`: it facilitates the transfer of fixed memory to disk.
- `-disk-dynamic-computing`: it facilitates the transfer of variable memory to disk.

Employing both parameters allows VFT4 to move data from memory to disk in such a way that very large datasets can be processed even on low-end servers with limited RAM. However, it is essential to consider that this feature may come at the cost of decreased computational performance. In this way, the performance of VFT4 is directly influenced by the amount of memory sent to the disk. Hence, it is advisable, whenever possible, to compute by transferring only fixed memory to the disk. This approach strikes a delicate balance between memory consumption and computational efficiency, optimizing the overall performance of the application.

Optimized Memory Consumption

VFT3 has been implemented using C++, which introduces additional memory overhead compared to the C implementation of FT2. Moreover, its more efficient utilization of threads leads to an increase in memory consumption as the number of threads grows. This increase is primarily caused by the replication of data structures, synchronization mechanisms, stack space for each thread, and caching effects, among other factors.

VFT4, on the other hand, has been redesigned to minimize the use of objects in memory-intensive sections and to release memory as soon as it is no longer needed. Consequently, in sequential execution, the memory requirements of VFT4 are even lower than those of FT2.

Additionally, to reduce the overhead introduced by each thread, VFT4 optimizes the storage of common temporary data, ensuring that it is stored only once. This significantly minimizes the overhead associated with multithreading, resulting in more efficient parallel execution compared to VFT3.

Other optimizations and functionalities

- *Support for New and Compressed Formats*: VFT3 and FT2 are limited to supporting the FASTA and Phylip formats, both stored as plain text. In contrast, VFT4 has extended its support to include the widely used Nexus and FASTQ formats. The Nexus format allows for storing sequences and the initial tree within a single file. Additionally, it is common for datasets downloaded from internet repositories to be compressed in formats such as `.gz` or `.bz`. Previously, it was required to manually decompress these files before using them. However, VFT4 can directly read compressed sequences in compatible formats, thanks to the integration of the Zlib and libBZ2 libraries. This approach saves time by removing the need for manual file decompression, resulting in improved performance and avoiding the additional effort of reading larger uncompressed files from the disk.

- *Broader Compatibility*: VFT4 is a versatile tool that supports Linux, Windows, and macOS, including Windows executables. It is also conveniently available in the Bioconda package repository [13], making it easily accessible to the bioinformatics community. Furthermore, for macOS users, it is also available as a MacPorts package [14]. Finally, for Linux users, VFT4 is included as a package in all Debian Linux distributions, simplifying its installation and integration into various computing environments.

- *Better Compilation Support*: The compilation process has been enhanced by adding support for new compilers, such as *clang*, and incorporating new features like AVX512 in Windows builds. The code has been optimized to comply with the latest compiler standards. Furthermore, parallel compilation has been implemented to accelerate the overall compilation time.

Performance evaluation

Next, we present experimental results that clearly demonstrate the superior performance of our tool, VFT4, compared to its predecessors, VFT3 and FT2. We have evaluated the following aspects: running time, memory consumption, CPU usage and topological accuracy.

For the evaluation, we selected three very large datasets with varying number of taxa and alignment lengths. Please refer to Table 1 for specific details about these datasets. It is important to note that only unique sequences were considered when constructing the trees. To ensure test reproducibility, we have included information about the parameters used to build the trees for each dataset:

- *Large*: `-spr 4 -gamma`
- *Very-Large*: `-nt -gamma -gtr`
- *Ultra-Large*: `-nt -gamma -gtr`

The arguments have the following meanings:

- `-nt`: Nucleotides alignment used as input.

Table 1. Characteristics of the datasets used in the experimental evaluation. Information obtained using *BigSeqKit* [15].

Dataset label	File name	Format	Type	Sequences	Unique Sequences	Length
Large	*selo3n.masked*	FASTA	AA	331,550	274,401	1,287
Very-Large	*gg_12_10_aligned*	FASTA	DNA	1,075,170	858,234	7,682
Ultra-Large	*1-million-taxon-run1*	NEXUS	DNA	1,000,000	989,109	21,946

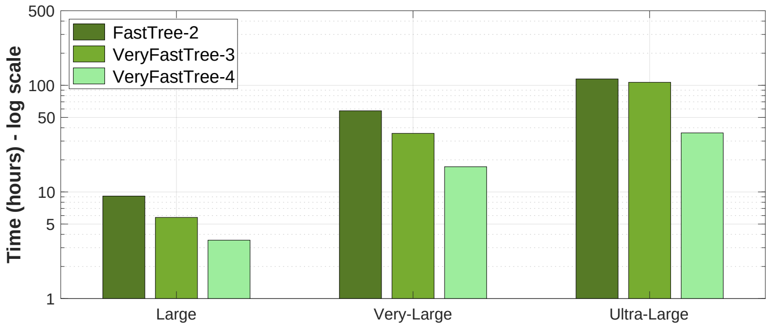

Figure 4. Running times of FT2, VFT3 and VFT4 for building the trees using single precision arithmetic and different datasets.

- `-spr`: Sets the number of rounds of subtree-prune-regraft (SPR) moves (default value is 2).
- `-gtr`: Utilizes the generalized time-reversible (GTR) model of nucleotide substitution.
- `-gamma`: After optimizing the tree with a fixed rate for each site (the CAT model), VFT (and FT2) will rescale the tree to optimize the Gamma20 likelihood [5].

Experiments were conducted using one server with two 32-core Intel Xeon Ice Lake 8352Y @2.2GHz processors and 512 GB of RAM. This server is part of a cluster installed at CESGA (Galicia Supercomputing Center, Spain) [16] running Rocky Linux v8.4 (kernel v4.18.0). We have used in the performance comparison the following tools and versions: Fast-Tree v.2.1.11 [17] , VeryFastTree v3.0 (our previous version) and VeryFastTree v4.0.3.

Running Times. First, we show in Figure 4 the running times when building the trees using single precision. We must highlight that, even if single precision is selected in VFT4 and FT2, many intermediate calculations such as vector reductions are performed with double precision to minimize errors. In the case of the *Large* dataset, VFT4 outperforms FT2 and VFT3, achieving speed improvements of 2.6× and 1.6× respectively, resulting in an execution time of just 3.5 hours. When inferring the phylogenetic tree from the *Very-Large* dataset, VFT4 completes the task in 17.2 hours, whereas FT2 requires 57.7 hours and VFT3 takes 35.4 hours. Finally, dealing with the *Ultra-Large* dataset, VFT4 is able to build the tree in 35.8 hours (i.e., 1.5 days). Note that the time required by FT2 and VFT3 increases noticeably to 4.8 and 4.5 days, respectively. In other words, VFT4 is 3.2× and 3× faster than FT2 and VFT3.

On the other hand, Figure 5 presents the running times for tree construction but using double precision arithmetic. In this case, for the *Large* dataset, VFT4 is 6.9× and 2.4× faster than FT2 and VFT3, respectively, reducing the execution time to less than 3 hours. Note that VFT4 builds the tree faster when using double precision than considering single precision (2.8h versus 3.5h). This is caused by the good behavior of the vectorization strategies used by VFT4 when considering protein alignments. On the other hand, inferring the phylogenetic tree from the *Very-Large* dataset using VFT4 takes 18 hours, while FT2 requires 61.7 hours and VFT3 41.3 hours. As a result, VFT4 is again the fastest tool. In conclusion, when dealing with the *Ultra-Large* dataset, VFT4 can complete the tree-building process within 41.2 hours, or 1.7 days. Notably, the time required by FT2 and VFT3 increases significantly to 5.3 and 4.6 days, respectively. To put it simply, VFT outperforms FT2 and VFT3 in speed by 3.1 times and 2.7 times, respectively.

As mentioned in the Introduction, in addition to FT2 and VFT, there are other state-of-the-art tools for ML tree estimation, with RAxML and IQ-TREE being the most commonly used by the scientific community. However, they are limited in their ability to process datasets containing a large number of sequences due to their extensive running times. To validate the observations made in previous works [4], we assessed both tools, RAxML-NG v.1.2.0 and IQ-TREE v.2.1.3, using our *Large* dataset. Since RAxML-NG can be executed on a cluster, we conducted experiments with this tool using four computing nodes instead of just one server. We manually constrained the experiments to a maximum running time of one week. Both tools were unable to construct the tree within that time frame. It is worth noting that VFT4 can estimate the tree for the *Large* dataset in approximately 3 hours (see Figures 4 and 5). This confirms that both RAxML and IQ-TREE are not well-suited for datasets containing a very large number of sequences.

Therefore, to the best of our knowledge, these results establish VFT4 as the fastest tool in the state-of-the-art for ML phylogeny estimation. But moreover, VFT4 allows the processing of massive datasets that would otherwise be intractable or would require excessively high computing times.

Memory consumption and CPU usage. In the previous section, we pointed out how the more effective use of threads results in higher memory consumption as the number of threads increases. VFT4 addresses this issue by optimizing the storage of commonly used temporary data, ensuring it is stored only once. As a result, the overhead associated with multithreading is significantly reduced, leading to a more efficient parallel execution compared to VFT3.

Figure 6 displays the CPU usage and memory consumption for all the considered tools when building the tree from the *Ultra-Large* dataset using single precision arithmetic. The purpose of this illustration is to showcase the memory optimizations implemented in VFT4. Each time step in the graphs corresponds to 3,600 seconds. It can be observed that the maximum memory consumed by VFT4 is a bit higher with respect to FT2, 272 GB and 228 GB, respectively. This increase is attributed to a more efficient utilization of threads. It is important to note that VFT4 extensively utilizes parallelism, employing the maximum available number of threads (64) for most of the time. In contrast, FT2 is constrained to using only a few threads, as indicated by the blue lines in the figures. As a consequence, VFT4 requires some extra memory for multithreading, but it comes with

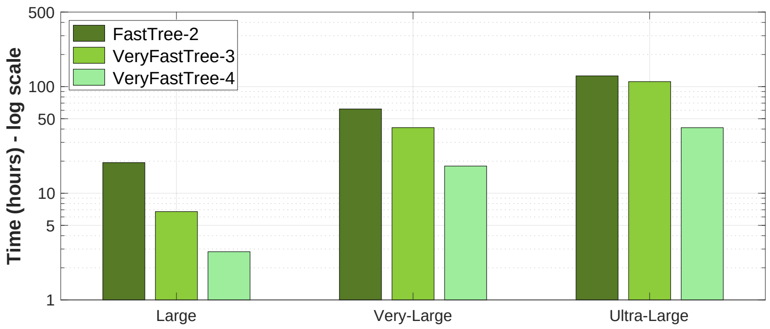

Figure 5. Running times of FT2, VFT3 and VFT4 for building the trees using double precision arithmetic and different datasets.

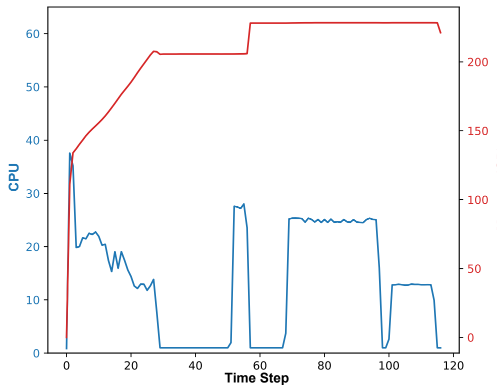

(a) FT2

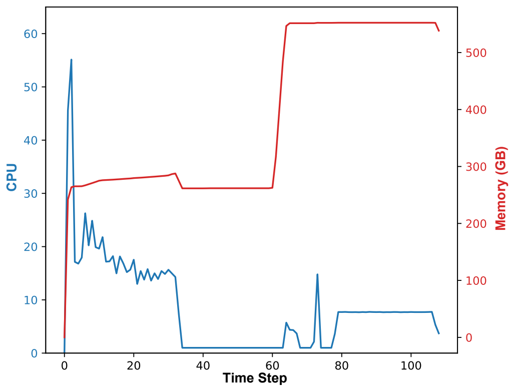

(b) VFT3

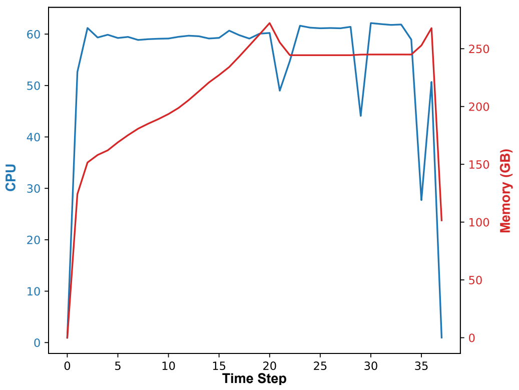

(c) VFT4

Figure 6. CPU usage (# of cores) and memory consumption when building the single precision trees using as input the *Ultra-Large* dataset. Time step = 3,600 seconds.

the benefit of a remarkable decrease in the running time. On the other hand, VFT4 significantly reduces memory consumption compared to VFT3. In particular, VFT3 requires twice the maximum memory used by VFT4. Furthermore, it also demonstrates greater efficiency in terms of parallelism.

As we pointed out previously, VFT4, like FT2, use sequence profiles of internal nodes of the tree to implement Neighbor-Joining instead of storing a distance matrix. Typically, as it was stated in [12], a distance matrix for an alignment with N sequences, L sites, and a different characters in the alphabet, requires $O(N^2)$ space. However, the upper bound of memory space required by VFT and FT2 is $O(NLa)$. Each profile comprises a frequency vector for each position, and the weighted average of its children's profiles.

If we take a look in detail, VFT4 requires 272 GB of memory to process the *Ultra-Large* dataset (see Figure 6(c)). Its corresponding parameters are: $N = 989,109$, $L = 21,946$, and $a = 4$ (nucleotides). As a consequence, taking into account that the resulting tree contains $2N$ nodes and single precision uses 4 bytes, the memory required by the profiles is the sum of the weights and frequencies for each node in the tree:

- Weights require exactly $2N \times L \times 4$ bytes = $(2 \times 989,109) \times 21,946 \times 4$ bytes = 173.65 GB.
- The memory of frequencies can be estimated as: $2N \times a \times g \times 4$ bytes, being 'g' the number of gaps in the sequence. Since 'g' is not a constant value and changes in each node as the topology of the tree evolves, an exact value for the memory space used cannot be provided. In any case, most of the difference between the total memory used by VFT4 (272 GB) and the memory required only by the weights of the profiles (173.65 GB) is due to the frequencies.

Disk computing. In many cases, researchers are faced with limited

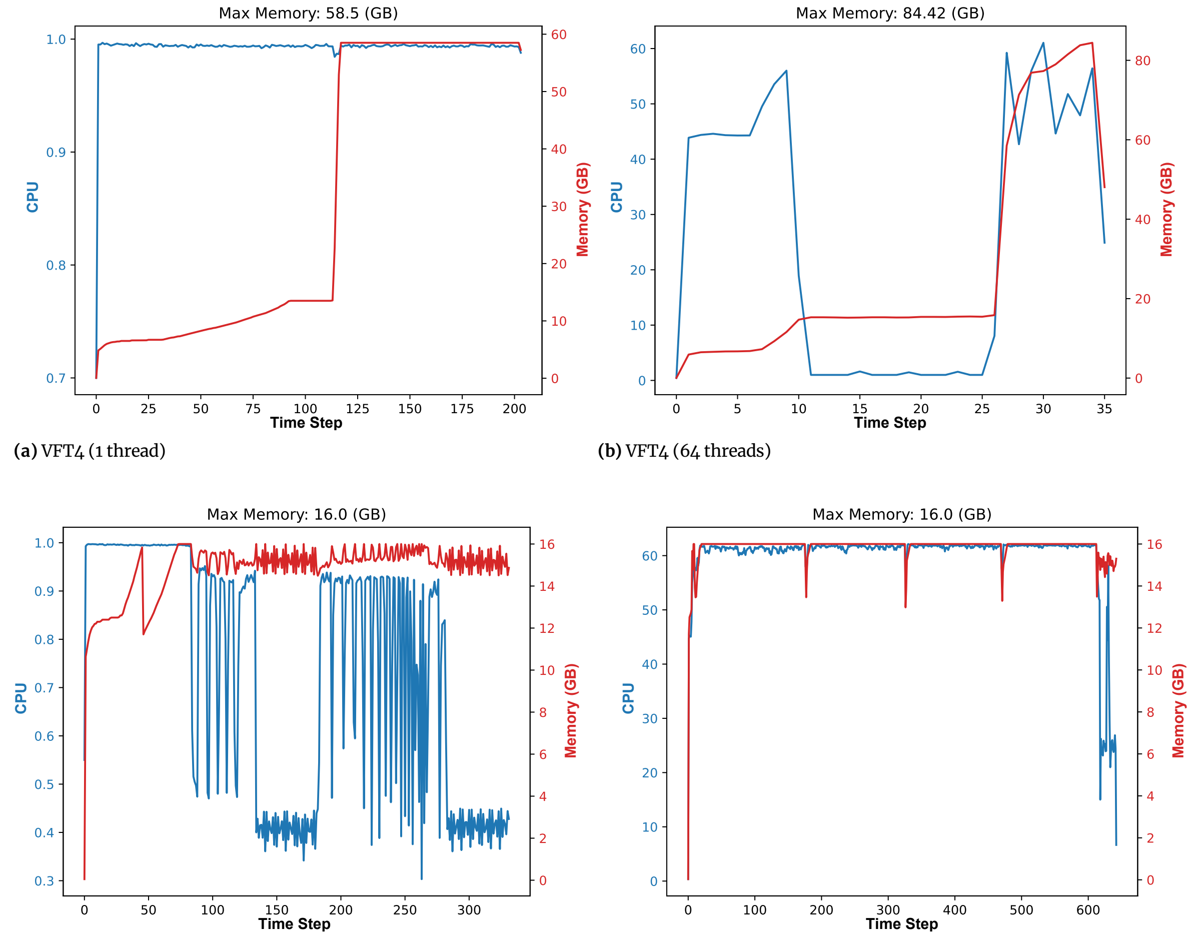

(a) VFT4 (1 thread)

(b) VFT4 (64 threads)

(c) VFT4 with disk-computing enabled (1 thread)

(d) VFT4 with disk-computing enabled (64 threads)

Figure 7. Effects of using disk computing when building the double precision trees using as input the *Large* dataset. Time step = 300 seconds.

computing resources, often having access only to low-end servers that possess a limited amount of memory. To deal with this issue, as was commented previously, we introduced *disk computing* in VFT4, a new feature that allows to offload static and dynamic memory to the disk with the aim of handling very large datasets even on small servers. The obvious drawback is an increase in the running times.

An example of the effects of disk computing can be found in Figure 7. Each time step in the graphs corresponds to 300 seconds. In particular, Figures 7(a) and 7(b) show the CPU usage and the memory footprint in a normal execution of VFT4 when processing the *Large* dataset considering double precision arithmetic, and 1 and 64 threads, respectively. In these cases, the maximum memory consumed were 58.5 GB (1 thread) and 84.42 GB (64 threads). Note that the spike in the memory consumption is caused by the computation and storage of the profiles at each node of the tree. On the other hand, Figures 7(c) and 7(d) also display the CPU usage and the memory footprint but using disk computing with 1 and 64 threads, and limiting the memory of the server to just 16 GB. It can be observed that thanks to this new feature, we can successfully build the tree on a small server. Considering 64 threads, for example, the maximum memory used is approximately 5.3× less compared to a normal execution. It may seem apparent, but without disk computing, the processing of datasets that surpass the available memory would be impossible. It would result in an out of memory error, which would be the case of using both FT2 and VFT3.

On the other hand, the VFT4 running time increases. Using 1 thread, from 16.8 to 27.5 hours, while using 64 threads from 2.8 to 53.4 hours. In other words, using disk computing on a 16 GB server is from 1.6× (1 thread) to 19× (64 threads) slower than the standard execution. An interesting observation is that the sequential execution with disk computing is faster than the corresponding execution with 64 threads. This is due to the limitations of disk and I/O bandwidth, which struggle to handle the extremely high number of requests generated when using 64 threads, leading to contention on the bus. Therefore, we recommend reducing the number of threads when utilizing the disk computing feature.

Topological Accuracy. In the literature, several studies have examined the performance of leading ML tools, such as RaxML, IQ-TREE, and FT2, regarding accuracy and computational efficiency with large datasets. Liu et al. [18] compared RAxML and FT2 using simulated datasets for single genes containing 1,000 or more sequences across various estimated alignments. They concluded that both methods exhibited similar topological accuracy. Lees et al. [19], in their exploration of ML heuristics through simulations, found RAxML and IQ-TREE to be comparable in accuracy, both outperforming FT2. Moreover, two studies [20, 21] evaluated FT2 and RAxML on datasets containing fragmentary sequences. They observed that FT2 performed less accurately than RAxML when handling alignments with a high proportion of fragmentary sequences. Overall, these studies have shown that IQ-TREE and RAxML are both very good ML heuristics with respect to ML scores, while all of the studies have shown that FT2 is indeed very fast, but it is so good at ML score optimization.

On the other hand, as we explained in our previous paper [6],

Table 2. Topological accuracies (in %) obtained by FT2, VFT3 and VFT4 using 5,000-sequences simulated protein alignments. Note that the parallel version of FT2 is non-deterministic, so minimum and maximum values are displayed between brackets.

Tool	COG438	COG583	COG596	COG642	COG1028	COG1309	COG2814	Average
VFT4 (level 3)	85.35	79.11	88.14	82.66	86.19	86.55	79.59	**83.94**
VFT4 (level 4)	85.43	79.13	87.72	82.19	86.37	87.29	79.92	**84.01**
VFT3	85.51	79.57	87.82	81.89	86.29	87.09	80.18	**84.05**
FT2	[85,85.83]	[79.21,79.91]	[88.04,88.78]	[82.11,82.95]	[86.30,87.03]	[86.19,87.59]	[79.06,80.66]	**[83.70,84.68]**

VFT is a highly tuned implementation of FT2 that keeps the phases, methods and heuristics used by FT2 to estimate the phylogenetic tree. For this reason, it is only necessary to compare the topological accuracies with respect to FT2 (and our previous version of VFT), since the works commented above already performed a thorough comparison between RAxML, IQ-TREE and FT2.

Similar to [5], we defined the topological accuracy as the proportion of splits in the true trees that are successfully recovered by each respective tool. This metric is the inverse of the topological Robinson-Foulds distance [22], normalized to a range between 0 and 1.

Our findings demonstrate that both VFT3 and VFT4 exhibit determinism while maintaining the same level of accuracy as FT2. To validate this, we assessed topological accuracy using 5,000-sequences simulated protein alignments [17], which were also employed in the original FT2 paper for the same purpose. Therefore, the true phylogeny is available. All simulations are based on trees from profile alignments of biological sequences and include variable rates across sites. The simulations are described in more detail in [23]. Trees were computed using 64 threads, double precision arithmetic and `-gamma -spr 4` parameters. All tools used the same seed for initializing the random number generator. The experimentation script, `treecmp.py`, is accessible in our repository. We show results for VFT4 considering two different levels of parallelism (i.e., *thread levels*). As we explained previously, level 3 performs in parallel all the computations but SPRs, while level 4 leverages the tree partitioning method to accelerate also SPR movements. For VFT3 and VFT4, topological accuracy values were computed by averaging seven measurements (one for each alignment in the dataset). Conversely, parallel executions of FT2 did not yield deterministic results. Consequently, we present a range of accuracy values. This range was derived by averaging the minimum and maximum values obtained from ten executions for each alignment. Accuracy results expressed in percentage are shown in Table 2.

Based on the results obtained, we conclude that VFT4 produces trees with an accuracy level within the same range as FT2. Additionally, minor differences were observed when using different thread levels. In particular, the most aggressive one in terms of parallelism, level 4, shows a slightly better behavior than level 3.

Commands interface

The new VeryFastTree version 4.0 was designed with extensive cross-platform compatibility, offering support for a variety of operating systems, including Linux, Windows, and macOS. This broad compatibility ensures that users can access and utilize the tool seamlessly across different computing environments. Windows users, in particular, benefit from the availability of executable versions of VFT4, simplifying the installation process and widening its user base. Additionally, for those in the bioinformatics field, VFT4 can be effortlessly located within the Bioconda package repository, streamlining both installation and integration into bioinformatics workflows. VFT4 is also available for macOS users as a MacPorts package. Finally, Linux users will appreciate that VFT4 is included as a package in all Debian Linux distributions, making installation straightforward and facilitating its integration into diverse computing setups.

Just like the previous version of VeryFastTree, it implements the same command interface than FT2. This means that the arguments behave exactly the same as in FT2. To check all these arguments, the *"-h"* or *"-expert"* option can be used. Consequently, to benefit from the performance advantages provided by VFT4, it is only necessary to replace the call to FT2 with a call to VFT4, using the same options.

On the other hand, VFT4 has its own extra arguments which have been grouped in the *Optimizations* section. These arguments are related to the parametrization of the different parallelization, vectorization and optimization strategies included in VFT4. Next we list and explain the new arguments available:

- `-threads [n]`
  It allows to specify the number of threads (n) used in the parallel execution. If this option is not set, the corresponding value will be obtained from the environment variable *OMP_NUM_THREADS*. This is the same approach followed by FT2. If $n = 1$, VeryFastTree behaves in the same way than FT2 compiled without the *-DOPENMP* flag.

- `-threads-level [level]`
  It allows to change the degree of parallelization.

  – If level is 0, VeryFastTree uses the same parallelization strategy as FT2 with some new parallel blocks.
  – If level is 1, VeryFastTree uses parallel blocks that require additional memory for computation.
  – If level is 2, VeryFastTree accelerates the rounds of ML NNIs using its tree partitioning method.
  – If level is 3 (default), VeryFastTree performs more computations without preserving sequential order.
  – If level is 4, VeryFastTree also accelerates the rounds of SPR steps using its tree partitioning method (it can only be used with datasets larger than $2^{maxSPRlength+2}$.

  Note: Each level includes the previous ones, and computation at level 2 and above is performed in a different tree traverse order, so the result may change.

- `-threads-mode [mode]`
  Changes the mode of parallelization.

  – If mode is 0, VeryFastTree uses non-deterministic parts, some inspired by FT2 but improved.
  – If mode is 1, VeryFastTree only uses deterministic parallelization.

  Since version 4.0, deterministic algorithms are at least faster than non-deterministic ones, making deterministic the preferred choice.

- `-threads-ptw [n]` (Partitioning Tendency Window)
  It sets the size of the partitioning tendency window used by the tree partitioning algorithm to determine when to stop searching. The window stores the last solutions and checks if a

better solution can be found. Increasing the value allows the algorithm to explore the tree deeper and potentially find better solutions. The default value is 50.

- `-threads-verbose`
  To show subtrees assigned to the threads and theoretical speedup, only with *verbose* > 0.

- `-double-precision`
  Use double precision arithmetic. Therefore, it is equivalent to compile FT2 with *-DUSE_DOUBLE*.

- `-ext [type]`
  It enables the vector extensions:

  – *AUTO*: (default) selects AVX2 when `-double-precision` is used and SSE3 otherwise. If one extension is not available, the previous level is used.
  – *NONE*: Operations are performed with the native programming language operators. In addition, loops are unrolled with the aim of providing hints to the compiler for applying some optimization (including vectorization).
  – *SSE3*: Arithmetic operations are performed using SSE3 vector intrinsics. Each instruction operates on 128 bit registers, which could contain four 32-bit floats or two 64-bit doubles.
  – *AVX*: Arithmetic operations are performed using AVX vector intrinsics. Each instruction operates on 256 bit registers, which could contain eight 32-bit floats or four 64-bits doubles.
  – *AVX2*: Similar to AVX, but some arithmetic operations are performed using additional AVX2 vector intrinsics not included in the AVX instruction set. Each instruction operates on 256 bit registers, which could contain eight 32-bit floats or four 64-bit doubles.
  – *AVX512*: Arithmetic operations are performed using AVX512 vector intrinsics. Each instruction operates on 512 bit registers, which could contain sixteen 32-bit floats or eight 64-bits doubles.

- `-disk-computing`
  If there is not enough available RAM to perform the computation, disk will be used to store extra data when it was not needed. Using disk to perform the computation will substantially increase the execution time.

- `-disk-computing-path [path]`
  Like *-disk-computing* but using a custom path folder to store data.

- `-disk-dynamic-computing`
  By default, disk computing only creates files associated with static data in RAM, which means that there is no significant impact on performance as long as there is available RAM. This option further reduces memory usage by storing dynamic data on disk. However, even if there is enough RAM, it will have a negative impact on performance due to the creation and deletion of files.

- `-seed [seed]`
  Set the seed for the random number generator. Default value is 1253.

- `-fastexp [implementation]`
  This option is used to select an alternative implementation for the exponential function (e^x), which has a significant impact on performance:

  – 0: (default) Use the *exp* function included in the built-in math library with double precision.
  – 1: Use the *exp* function included in the built-in math library with simple precision (not recommended together with *-double-precision* option).
  – 2: Use a very efficient and fast implementation to compute an accurate approximation of e^x using double precision arithmetic.
  – 3: Use a very efficient and fast implementation to compute an accurate approximation of e^x using simple precision arithmetic (not recommended together with *-double-precision* option).

Conclusions

In the field of bioinformatics research, phylogenies are of utmost importance. Regrettably, the search for the optimal phylogenetic tree imposes significant computational requirements, and most modern cutting-edge tools struggle to handle exceptionally large datasets in a timely manner.

In this work, we introduce the latest version of VeryFastTree, which incorporates numerous performance optimizations and new features. Experimental results establish VeryFastTree as the fastest tool in the state-of-the-art for ML phylogeny estimation. For instance, it is capable of processing massive datasets containing one million taxa in just 36 hours, which is several times faster than other tools. In this way, VeryFastTree enables the processing of datasets that would otherwise be intractable or require excessively high computing times. Despite its exceptional speed, it produces trees with an accuracy level comparable to that of FastTree-2. On the other hand, a noteworthy new characteristic of VeryFastTree is what we call *disk computing*, which allows the processing of extremely large datasets on low-end servers with limited memory resources.

Finally, we would like to emphasize that VeryFastTree can also serve as the foundation for building an initial tree, which can subsequently be optimized using the latest developments in online phylogenetics [24, 25] or be used as a first step of Disjoint Tree Mergers (DTMs) [4, 8, 9, 26].

Funding

This work was supported by MICINN [PLEC2021-007662, PID2022-137061OB-C22]; Xunta de Galicia [ED431G 2019/04, ED431F 2020/08, ED431C 2022/16]; and ERDF.

Availability of source code and requirements

- Project name: VeryFastTree
- Project home page: `https://github.com/citiususc/veryfasttree`
- BiotoolsID: `biotools:veryfasttree`
- RRID: SCR_023594
- Operating system(s): Linux, Windows and macOS
- Programming language: C/C++
- Other requirements: -
- License: GNU GPL-3.0

Availability of supporting data and materials

The datasets supporting the results of this article are available in:

- *Large* dataset was obtained from the FastTree-2 tool website.
- *Very-Large* dataset was obtained from the Greengenes database.
- *Ultra-Large* dataset was obtained from the Kim Lab for Computational Evolutionary Biology (University of Pennsylvania, USA)

repository.
- 5,000-sequences simulated protein alignments dataset was obtained from the FastTree-2 tool website.

Declarations

List of abbreviations

(DTMs) Disjoint Tree Mergers, (FT2) FastTree-2, (ML) maximum-likelihood, (MP) maximum-parsimony, (MSA) Multiple sequence alignment, (NNIs) Nearest-neighbor interchanges, (SPR) Subtree pruning and regrafting, (VFT3) first version of VeryFastTree, (VFT4) latest version of VeryFastTree introduced in this work.

Ethical Approval

Not applicable.

Consent for publication

Not applicable.

Competing Interests

The authors declare that they have no competing interests.

Funding

This work was supported by MICINN [PLEC2021-007662, PID2022-137061OB-C22]; Xunta de Galicia [ED431G 2019/04, ED431F 2020/08, ED431C 2022/16]; and European Regional Development Fund (ERDF).

Author's Contributions

César Piñeiro: Methodology, Software Development, Conducted Experiments, and Contributed to Writing.
Juan C. Pichel: Conceptualization, Methodology, Supervision, Writing and Revision.

Acknowledgements

Authors wish to thank CESGA (Galicia, Spain) for providing access to their supercomputing facilities.

References

1. Warnow T. Computational Phylogenetics: An Introduction to Designing Methods for Phylogeny Estimation. Cambridge University Press; 2017.
2. Kozlov AM, Darriba D, Flouri T, Morel B, Stamatakis A. RAxML-NG: a fast, scalable and user-friendly tool for maximum likelihood phylogenetic inference. Bioinformatics 2019;35(21):4453–4455.
3. Minh BQ, Schmidt HA, Chernomor O, Schrempf D, Woodhams MD, Von Haeseler A, et al. IQ-TREE 2: new models and efficient methods for phylogenetic inference in the genomic era. Molecular biology and evolution 2020;37(5):1530–1534.
4. Park M, Zaharias P, Warnow T. Disjoint tree mergers for large-scale maximum likelihood tree estimation. Algorithms 2021;14(5):148.
5. Price MN, Dehal PS, Arkin AP. FastTree 2 - Approximately Maximum-Likelihood Trees for Large Alignments. PLOS ONE 2010;5(3):1–10.
6. Piñeiro C, Abuín JM, Pichel JC. VeryFastTree: speeding up the estimation of phylogenies for large alignments through parallelization and vectorization strategies. Bioinformatics 2020;36(17):4658–4659.
7. Nelesen S, Liu K, Wang LS, Linder CR, Warnow T. DACTAL: divide-and-conquer trees (almost) without alignments. Bioinformatics 2012;28(12):i274–i282.
8. Molloy EK, Warnow T. TreeMerge: a new method for improving the scalability of species tree estimation methods. Bioinformatics 2019;35(14):i417–i426.
9. Smirnov V, Warnow T. Unblended disjoint tree merging using GTM improves species tree estimation. BMC genomics 2020;21:1–17.
10. Heath MT. A tale of two laws. The International Journal of High Performance Computing Applications 2015;29(3):320–330.
11. Babel L, Kellerer H, Kotov V. The k-partitioning problem. Mathematical Methods of Operations Research 1998;47(1):59–82.
12. Price MN, Dehal PS, Arkin AP. FastTree: computing large minimum evolution trees with profiles instead of a distance matrix. Molecular biology and evolution 2009;26(7):1641–1650.
13. Piñeiro C, Veryfasttree; 2023. Bioconda package. https://anaconda.org/bioconda/veryfasttree [Online; accessed 16 October 2023].
14. Piñeiro C, Veryfasttree; 2023. MacPorts package. https://ports.macports.org/port/veryfasttree/ [Online; accessed 16 October 2023].
15. Piñeiro C, Pichel JC. BigSeqKit: a parallel Big Data toolkit to process FASTA and FASTQ files at scale. GigaScience 2023;12:giad062.
16. CESGA (Galician Supercomputing Center) - Computing Infrastructures;. https://www.cesga.es/en/infrastructures/computing/ [Online; accessed 16 October 2023].
17. Price MN, FastTree;. http://www.microbesonline.org/fasttree/ [Online; accessed 16 October 2023].
18. Liu K, Linder CR, Warnow T. RAxML and FastTree: Comparing Two Methods for Large-Scale Maximum Likelihood Phylogeny Estimation. PLOS ONE 2011;6(11):1–11.
19. Lees JA, Kendall M, Parkhill J, Colijn C, Bentley SD, Harris SR. Evaluation of phylogenetic reconstruction methods using bacterial whole genomes: a simulation based study. Wellcome open research 2018;3.
20. Smirnov V, Warnow T. Phylogeny estimation given sequence length heterogeneity. Systematic biology 2021;70(2):268–282.
21. Sayyari E, Whitfield JB, Mirarab S. Fragmentary gene sequences negatively impact gene tree and species tree reconstruction. Molecular biology and evolution 2017;34(12):3279–3291.
22. Huerta-Cepas J, Serra F, Bork P. ETE 3: Reconstruction, Analysis, and Visualization of Phylogenomic Data. Molecular Biology and Evolution 2016;33(6):1635–1638.
23. Tatusov RL, Natale DA, Garkavtsev IV, Tatusova TA, Shankavaram UT, Rao BS, et al. The COG database: new developments in phylogenetic classification of proteins from complete genomes. Nucleic acids research 2001;29(1):22–28.
24. Ye C, Thornlow B, Hinrichs A, Kramer A, Mirchandani C, Torvi D, et al. matOptimize: a parallel tree optimization method enables online phylogenetics for SARS-CoV-2. Bioinformatics 2022;38(15):3734–3740.
25. Turakhia Y, Thornlow B, Hinrichs A, McBroome J, Ayala N, Ye C, et al. Pandemic-scale phylogenomics reveals the SARS-CoV-2 recombination landscape. Nature 2022;609(7929):994–997.
26. Zhang QR, Rao S, Warnow T. New absolute fast converging phylogeny estimation methods with improved scalability and accuracy. In: 18th International Workshop on Algorithms in Bioinformatics (WABI) Schloss Dagstuhl-Leibniz-Zentrum fuer Informatik; 2018. .
