# Supplementary material for: Efficient phylogenetic tree inference for massive taxonomic datasets: harnessing the power of a server to analyze 1 million taxa
Source: Gigascience. 2024 Aug 8;13:giae055. doi: 10.1093/gigascience/giae055 (PMC11308190; doi:10.1093/gigascience/giae055)
Supplement: giae055_Response_to_Reviewer_Comments_Original_Submission [file giae055_response_to_reviewer_comments_original_submission.pdf]

Author's Response To Reviewer Comments

Efficient phylogenetic tree inference for massive taxonomic datasets: harnessing the power of a server to analyze one million taxa
César Piñeiro and Juan C. Pichel

We would like to thank the reviewers and editors for their insightful comments and suggestions about the paper. All the changes in the revised manuscript are highlighted in red. Detailed responses to reviewers are given below.

——

Reviewer #1: This paper presents VeryFastTree version 4, an update of the VeryFastTree method published initially in 2020.
The new version features an advanced parallelization scheme that results in utilizing the maximum number of threads over nearly the full runtime of the program. Together with memory and other technical improvements, this makes VFT4 the fastest ML tree builder available, as the authors demonstrate in benchmarks. VFT4 also handles compressed file formats and features a disk computing functionality that stores data on the disk instead of RAM. For users with limited RAM machines, the latter feature enables them to compute trees, which would otherwise not be possible without upgrading RAM.

###################
###################

The paper is well written and I only have minor suggestions:

1) The abstract is very short and does not mention the new features build in VFT4. The features could be briefly mentioned (for those readers that don't read the full text).

****RESPONSE*****

Following the suggestion of the reviewer, we have extended the abstract information in the revised manuscript with the aim of including briefly some of the new features of VFT4 (page 1).

###################
###################

2) It would be helpful to add a figure that illustrates the new parallelization scheme.
For example, the penalty parameter and parent / uncle swap is difficult to understand without seeing an illustration, especially because the penalty parameter is important for the new tree partitioning method.

****RESPONSE*****

We have significantly revised and expanded the section detailing the parallelization strategy of VFT4, spanning pages 2, 3, and 4 of the manuscript. These revisions include the incorporation of two new figures, one of which serves to illustrate the tree partitioning process and the role of the penalty value (Figure 1, page 2). A pseudocode explaining the tree partitioning algorithm was also included (Figure 2, page 3).

###################
###################

3) Why is level 3 and not level 4 the default level? Level 4 is faster and the authors mention that level 4 produces slightly higher accuracy. Is there a downside to level 4 parallelization?

****RESPONSE*****

Level 3 performs in parallel all the computations but SPRs, while Level 4 leverages the tree partitioning method to accelerate also SPR movements. However, the parallelization of the SPR only pays off when the number of alignments is high. In other words, when the tree is large enough. For this reason, Level 3 is chosen by default as the best starting option for the users.

We have clarified this question in the revised manuscript (page 4).

###################
###################

4) VFT4 is non-deterministic, which is great. My understanding is that the repeated runs of VFT4 with the same parameters result in identical results. However, how can users tell VFT4 to introduce variation in the tree space search? Is there something like a seed parameter that initializes a random number generator? If so, the user could test if different seeds result in similar or identical trees.

****RESPONSE*****

The reviewer is right. There is an argument to set the seed for the random number generator: "-seed n". Its default value is 1253. Now this option is explained in the "Commands interface" section of the revised text (page 10), and it was specified that accuracies for all the tools were computed using the same seed (page 9). Since the behavior observed for VFT3, VFT4 and FT2 using different seeds is equivalent to the one detailed in Table 2, we only provide accuracy results for one seed.

———

Reviewer #2: The paper presents VFT4 - an upgrade version of the authors' own tool VFT3. The new updates as described in the paper include the application of SPR for exploring the tree space as well as the support of new file formats (nexus and compressed files). The authors mainly compare the performance of VFT4 with VFT3 and FT2. My main concern with this manuscript is that there is insufficient detail on the new features of VFT4, often times paragraphs read too vague to understand. This of course does not mean the work should not be published; on the contrary I believe improvements to phylogenetic inference are important but the paper requires a substantial revision to explain its functionality, the concepts involved as well as experiments to demonstrate/evaluate its accuracy.

Please find below a couple more specific comments:

###################
###################

* The manuscript does not include any information regarding the scoring function used for ranking phylogenies. Does the tree search aim to find the maximum likelihood estimates of the parameters and the MLE tree, or is the search based on parsimony (e.g. the number of changes in the tree) or a combination of both approaches? The authors should clarify this.

****RESPONSE*****

As it was explained in [1], VFT preserves the phases, methods and heuristics used by FT2 to estimate the phylogenetic tree. As a consequence, VFT (and FT2) uses a method based on maximum-likelihood (ML) to estimate phylogenetic trees. The maximum likelihood method seeks to find the tree topology and branch lengths that maximize the likelihood of the observed sequence data given the evolutionary model and parameters. This approach accounts for the probabilities of different evolutionary events (such as substitutions, insertions, and deletions) and attempts to find the most likely evolutionary history given the data and model assumptions.

Therefore, VFT does not use a parsimony-based approach. While parsimony is a valid method for phylogenetic inference, VFT emphasizes maximum likelihood estimation due to its efficiency and scalability for large datasets.

This information is included in the Introduction of the revised manuscript that enumerates some of the most important state-of-the-art tools for maximum-likelihood phylogeny estimation (page 1).

[1] Piñeiro C, Abuín JM, Pichel JC. VeryFastTree: speeding up the estimation of phylogenies for large alignments through parallelization and vectorization strategies. Bioinformatics 2020;36(17):4658–4659.

###################
###################

Also, based on some comments on "parent" and "uncle" nodes, it's unclear whether the program operates on rooted or unrooted trees. While recognizing that such information might have been described in prior publications, it's important for each manuscript to stand independently in this regard.

****RESPONSE*****

VFT uses rooted trees. Now this information was included in the revised text (page 2, "Tree partitioning" subsection).

###################
###################

* The manuscript needs a thorough discussion on how the correctness of results is assessed. Have simulation studies been conducted to evaluate the performance of the algorithm? Are the results compared against known phylogenies or validated against other phylogenetic software. If such comparisons are made, please specifying the software packages and datasets used for validation. To establish the reliability of VFT4, it would be advantageous to perform simulation studies using a set of datasets generated from real data. This approach would allow for the assessment of how frequently the program converges to the true phylogeny . Furthermore, conducting empirical analyses and comparing the results obtained from VFT4 with established software packages such as RAxML or IQ-TREE would instill confidence in the program's accuracy and robustness.

****RESPONSE*****

Correctness:

In the literature, several studies have examined the performance of leading ML tools, such as RaxML, IQ-TREE, and FT2, regarding accuracy and computational efficiency with large datasets. Liu et al. [1] compared RAxML and FT2 using simulated datasets for single genes containing 1000 or more sequences across various estimated alignments. They concluded that both methods exhibited similar topological accuracy. Lees et al. [2], in their exploration of ML heuristics through simulations, found RAxML and IQ-TREE to be comparable in accuracy, both outperforming FT2. Moreover, two studies [3,4] evaluated FT2 and RAxML on datasets containing fragmentary sequences. They observed that FT2 performed less accurately than RAxML when handling alignments with a high proportion of fragmentary sequences. Overall, these studies have shown that IQ-TREE and RAxML are both very good ML heuristics with respect to ML scores, while all of the studies have shown that FT2 is indeed very fast, but it is not very good at ML score optimization.

On the other hand, as we explained in our previous paper [5], VFT is a highly tuned implementation of FT2 that keeps the phases, methods and heuristics used by FT2 to estimate the phylogenetic tree. For this reason, it is only necessary to compare the topological accuracies with respect to FT2 (and our previous version of VFT), since the previous works performed a thorough comparison between RAxML, IQ-TREE and FastTree-2. This study was shown in the original paper in page 6, concluding that VFT4 produces trees with an accuracy level within the same range as FT2. We have used 5,000 sequences simulated protein

alignments, which were also employed in the original FT2 paper for the same purpose. Therefore, the true phylogeny is available. All simulations are based on trees from profile alignments of biological sequences and include variable rates across sites. The simulations are described in more detail in [6].

Now we have included the above information in the revised manuscript (page 8 - "Topological accuracy" subsection). FT2 and VFT versions are detailed in page 6. We have also added five new references to the bibliography. In addition, the dataset containing the 5,000 sequences simulated protein alignments used in the topological accuracy experiments is now included in the "Availability of supporting data and materials" section (page 11).

[1] Liu, K., Linder, C.R., Warnow, T. RAxML and FastTree: Comparing two methods for large-scale maximum likelihood phylogeny estimation. PLoS ONE 2011, 6, e27731.
[2] Lees, J.A., Kendall, M., Parkhill, J., Colijn, C., Bentley, S.D., Harris, S.R. Evaluation of phylogenetic reconstruction methods using bacterial whole genomes: A simulation based study. Wellcome Open Res. 2018, 3, 33.
[3] Smirnov, V., Warnow, T. Phylogeny estimation given sequence length heterogeneity. Syst. Biol. 2021, 70, 268–282. \\
[4] Sayyari, E., Whitfield, J.B., Mirarab, S. Fragmentary gene sequences negatively impact gene tree and species tree reconstruction. Molecular Biology and Evolution. 2017, 34, 3279–3291.
[5] Piñeiro C, Abuín JM, Pichel JC. VeryFastTree: speeding up the estimation of phylogenies for large alignments through parallelization and vectorization strategies. Bioinformatics 2020;36(17):4658–4659.
[6] Tatusov, R.L., Natale, D.A., Garkavtsev, I.V., Tatusova, T.A., Shankavaram U.T., et al. The COG database: new developments in phylogenetic classification of proteins from complete genomes. Nucleic Acids Res. 2001, 29: 22–8.

Running times:

As we mentioned, besides FT2 and VFT, there exist other state-of-the-art tools for ML tree estimation, notably RAxML and IQ-TREE, widely embraced by the scientific community. However, they are limited in their ability to process datasets containing a large number of sequences due to their extensive running times. To validate the observations made in previous works, we evaluated RAxML-NG v.1.2.0 and IQ-TREE v.2.1.3 using our Large dataset. Since RAxML-NG can be executed on a cluster, we conducted experiments with this tool using four computing nodes instead of just one server. We manually constrained the experiments to a maximum running time of one week. Both tools were unable to construct the tree within that time frame. It is worth noting that VFT4 can estimate the tree for the Large dataset in approximately 3 hours. This reiterates the unsuitability of RAxML and IQ-TREE for handling datasets with a substantial number of sequences. This information can be found in the revised manuscript (page 6).

###################
###################

* I find it hard to believe that single precision is beneficial for trees of that size (even for the Large dataset). Trees of such height will inevitably suffer from underflow when calculating conditional probabilities. How do you account for that? Even if some sort of numerical scaling is employed (e.g. adding a constant when reaching a threshold) I assume it is happening so often that it negates any benefit over using double precision (with scaling). Some discussion on this would be very useful.

****RESPONSE*****

Certainly, VFT implements numerical scaling to mitigate underflow, primarily through normalization techniques, adding constants or logarithmic values. Nonetheless, a minimum threshold is established to identify closely-related sequences, prompting user notification regarding calculation challenges and encouraging the adoption of double precision. Even if single precision is selected, many intermediate calculations such as vector reductions are performed with double precision to minimize errors, although final results influencing conditional probabilities are reverted to single precision.

This information is now included in the revised text (page 6).

###################
###################

* "New parallel regions"
The discussion on parallelization of tree traversals doesn't sound right. The overhead involved with traversing a tree structure, even with millions of nodes, is negligible compared to evaluating the scoring function (likelihood/parsimony/etc). My understanding is that a tree traversal involves generating an ordering for the tree nodes (e.g. postorder). It appears the authors may be referring to parallelizing the entire process of topology and evaluation, but this requires clarification. Moreover, the purpose of tree partitioning and the utilization of breadth-first traversal in this context lacks explanation. The text suggests that the tree is divided into disjoint graphs so that threads can work independently (although this "work" is not explained), yet it still happens that threads may need to operate outside of their partitioned graph, and a penalty parameter is used to avoid this.
Furthermore, it's unclear how topological changes (if that's what the authors describe) and branch lengths optimizations are executed in parallel on independent subtrees. Particularly, if the likelihood function is used for scoring, it is expected that changes necessitate re-evaluation of the likelihood by updating the conditional probabilities along the path to the root, assuming the remaining part of the tree is fixed (which I understand is not the case if you are working on different parts in parallel). This is not described in the manuscript.

****RESPONSE*****

Responding to several queries from the reviewer regarding the clarification and enhancement of the explanation of the parallelization process in VFT4, we have extensively revised and expanded the corresponding section, which now spans pages 2, 3, and 4 of the manuscript. These revisions include the introduction of two new figures (Figures 1 and 3), the inclusion of one pseudocode (Figure 2), and the addition of three new references [10-12] to aid in understanding.

###################
###################

* Finally, the section ends with the statement "On the other hand, in the parallel traverse approach, nodes at the same height level are computed simultaneously", which lacks clarity regarding what is computed and the methodology employed. The remaining part of the paragraph contains several undefined terms, such as "initial profiles" further exacerbating the confusion.

****RESPONSE*****

We have completely rewritten the text about the parallel breadth-first traversal (new subsection in page 4 of the revised manuscript): "Tree partitioning enables parallel processing of a single tree by assigning disjoint subtrees to each thread, allowing them to compute independently and without interference. However, when an operation has no dependencies and solely modifies the node being computed, creating subtrees is inefficient, as each node can be directly assigned to a thread. So, starting from the deepest nodes of the tree, each thread handles a portion of the nodes and computes them simultaneously, following a breadth-first traversal pattern, which involves visiting all nodes at a given depth level before moving on to the next level. Once finished with that level, the upper levels are distributed until the root of the tree is reached. This approach is specifically utilized in the following operations: computing initial profiles, recomputing profiles, recomputing ML profiles, computing tree length, and computing the likelihood for each site."

On the other hand, FT2 and VFT implements Neighbor-Joining by storing profiles for the internal nodes in the tree instead of storing a distance matrix with the aim of reducing the memory footprint. This information was detailed in the original FastTree papers [1,2]. Each profile includes a frequency vector for each position, and the weighted average of its children’s profiles. FT2 and VFT use these profiles to compute the distances between internal nodes in the tree and also the total distance from a node to all other nodes. Now we have clarified this in the paper (page 4) and we also refer to the FT papers for

details.

[1] Price MN, Dehal PS, Arkin AP. FastTree: computing large minimum evolution trees with profiles instead of a distance matrix. Molecular biology and evolution 2009;26(7):1641–1650.
[2] Price MN, Dehal PS, Arkin AP. FastTree 2 - Approximately Maximum-Likelihood Trees for Large Alignments. PLOS ONE 2010;5(3):1–10.

###################
###################

To enhance clarity, I advise to replace this section with a more intuitive explanation of the parallelization strategy. This revised section should delineate what aspects of the program were parallelized, the previous state of the program and the benefits accrued from parallelization. A figure illustrating the parallelization would greatly enhance the presentation of the manuscript. Technical intricacies can be relegated to supplementary material for readers seeking detailed understanding.

****RESPONSE*****

As previously mentioned, in response to the reviewer's suggestion, we have undertaken substantial revisions and expansions to the section elucidating the parallelization strategy of VFT4 (pages 2, 3 and 4 of the revised manuscript). This includes a detailed explanation of the tree partitioning algorithm, the mapping of subtrees to threads, the proposed stopping criterion aimed at reducing the search space when seeking the best partitioning, and the parallel breadth-first traversal.

###################
###################

* Non-deterministic deprecation

This section lacks informative content and delves excessively into details without contextualization. To improve clarity, replace the sentence 'certain sections of the code perform synchronized simultaneous modifications using mutex (a mechanism for controlling access to shared resources)' with 'critical regions', a standard term in the context of parallelizations. If the reader is not familiar with such a term, they will certainly not understand concepts like 'mutex' either. Additionally, the paragraph discusses some critical region, which had a non-deterministic behaviour, became deterministic, then it evolved back to non-deterministic again, and now, in VFT4, it's changed again to determinstic, without describing its function, the nature of deterministic and non-deterministic actions within it, or the implications for speed and memory footprint. I don't think this paragraph provides any meaningful information to the reader (at least not to me), and perhaps consider either clarifying or deleting it.

****RESPONSE*****

After careful consideration, we recognize that the paragraph discussing determinism may not provide meaningful information to readers, especially without sufficient context or explanation of its implications. Given its technical nature, we have opted to remove this paragraph from the manuscript.

###################
###################

* Some principles appear ot be misused, particularly regarding memory categorization. On page 3, the authors classify program memory (RAM) into static, dynamic and computation categories. However, the term "static memory" traditionally refers to memory that is allocated at compile time, and which remains unchanged throughout program execution. It appears that all three memory types mentioned by the authors are part of dynamic memory, allocated during the program execution. The description suggests that some dynamically allocated memory is transferred to disk when not actively utilized, a process which is akin to swapping managed by the operating system. The rationale behind implementing manual memory swapping within the program raises questions, especially considering that modern operating systems

already employ sophisticated swapping mechanisms. Leveraging OS-managed swapping could obviate the need for custom memory management within the program, and thus the obvious question is why not just let the OS do the swapping when necessary?
Moreover, the manuscript lacks clarity regarding the specific contents of memory being swapped to disk. While the authors mention static and dynamic memory is subjected to swapping (in my terminology, any kind of memory allocated by the program at run-time), it remains unclear whether that encompasses things like intermediate likelihood/parsimony results at each node, which should be accessible by each thread (i.e. shared memory) and form the main bulk of memory scheme. Finally, I would imagine that the part of memory described as "computation" should be tiny compared to intermediate likelihood scores. Or do you refer to local NUMA memory as "computation"? In any case, describing the precise memory contents slated for swapping would be greatly appreciated by other software developers in the field.

****RESPONSE*****

The reviewer is right, as all memory types discussed in the text pertain to dynamically allocated memory. To alleviate any confusion, we have opted to rename these memory types and explicitly specify that we are exclusively referring to types of dynamic memory. First, fixed memory (previously static) refers to memory allocated at the start of the program, once the properties of the input dataset (such as the number of sequences, length, etc.) are established. For instance, fixed memory is used to store sequences and weights in each profile. Variable memory (previously dynamic) is the memory used to store the frequency vector in each profile (conditional probabilities) whose size changes dynamically and cannot be precisely predicted. Finally, computation memory refers to the requirements of temporary memory that are released after producing an intermediate result.

On the other hand, using swap space as an alternative when a process exceeds RAM capacity has important limitations and drawbacks. First, the swap partition has a fixed size, potentially leading to out-of-memory errors if exceeded. This is especially relevant when dealing with very large datasets, as is our case. Therefore, if the dataset exceeds the size of the swap, only an administrator would have the authority to expand the swap partition to prevent the job from being aborted. In addition, since all programs share the same swap space, excessive swapping by one program can adversely affect others, leading to contention for disk I/O resources and reduced responsiveness. In contrast, the mechanism used by VFT4 in "disk computing" is based on memory-mapped files, which offers better I/O performance due to the optimized kernel-level caching mechanisms and efficient block-based access. Note that when using memory mapping any available disk can be used to store data, avoiding the swap partition size limitation. Additionally, memory-mapped files are also controlled by the operating system, similar to swap space, and are not manually managed. This ensures efficient handling of data movement between memory and the disk, with the operating system making decisions based on system resources and requirements.

Finally, computation memory, even for huge datasets, remains significantly smaller in comparison to fixed and variable memories.

We have included and extended the above information in the "Disk computing" section (pages 4-5).

###################
###################

* Performance evaluation: please explain what the -spr 4 -gamma -gtr switches mean. I assume a GTR+Gamma model is used with a discretized gamma approximation for rate heterogeneity using 4 categories, but this should be stated explicitly. Also, explain what the -spr 4 switch does.

****RESPONSE*****

-> -nt option: Nucleotides alignment used as input.
-> -spr option: Set the number of rounds of subtree-prune-regraft (SPR) moves (default value is 2).
-> -gtr option: The generalized time-reversible (GTR) model of nucleotide substitution is used.
-> -gamma option: VFT (and FT2) will report the likelihood under the discrete gamma model with 20 rate categories ("Gamma20"). More precisely, after optimizing the tree with a fixed rate for each site (the CAT

model), VFT (and FT2) will rescale the tree to optimize the Gamma20 likelihood. That is explained and justified in the original paper of FT2 [1].

Now this information was included in the revised manuscript (pages 5-6).

[1] Price MN, Dehal PS, Arkin AP. FastTree 2 - Approximately Maximum-Likelihood Trees for Large Alignments. PLOS ONE 2010;5(3):1–10.

###################
###################

* Figure 1 and Figure 2 do not seem to match the text. I think the third cluster of bars (Ultra-large dataset) should be swapped between the figures?

****RESPONSE*****

There was an error in the Y axis (Time) in Figure 2 (double precision running times). Now this was solved in the revised text (now Figure 5, page 7). In addition, we have included the following explanation about the lower time obtained with the large dataset and double precision compared to the time observed for single precision (page 6): "Note that VFT4 builds the tree faster when using double precision than considering single precision (2.8h versus 3.5h). This is caused by the good behavior of the vectorization strategies used by VFT4 when considering protein alignments."

###################
###################

* Figure 3. Assuming VFT4 involves ML estimation using GTR-G4 and minimal site compression due to the amount of sequences, the memory requirement for the Ultra-Large dataset would be roughly ~ 1.4 TB [inner nodes * sites * states * rates * sizeof(float) = (989109-2)*21946*4*4*4] and that's accounting for only the conditional probabilities at inner nodes. Similarly, figs 4ab shows memory usage for the "Large" dataset using double precision. Based on my calculations the expected memory required for storing the conditional probabilities should be (274401-2)*1287*20*4*8 ~= 230 GB (assuming no site compression), yet the plotted max memory usage at a time is at most ~ 80 GB with 75\% of time being at ~ 10GB. This is a huge discrepancy and should be explained/discussed whether VFT4 is doing something very smart or if there is a huge amount of gappy or identical sites affecting memory use.

****RESPONSE*****

As we pointed out previously, VFT4, like FT2, use sequence profiles of internal nodes of the tree to implement Neighbor-Joining instead of storing a distance matrix. Typically, as it was stated in [1], a distance matrix for an alignment with 'N' sequences, 'L' sites, and 'a' different characters in the alphabet, requires $O(N^2)$ space. However, the upper bound of memory space required by VFT4 and FT2 is $O(NLa)$. Each profile comprises a frequency vector for each position, and the weighted average of its children’s profiles.

If we consider the Ultra-Large dataset, whose required memory is 272 GB, its corresponding parameters are: N = 989,109, L = 21,946, and a = 4 (nucleotides). As a consequence, taking into account that the resulting tree contains 2N nodes and single precision uses 4 bytes, the memory required by the profiles is the sum of the weights and frequencies for each node in the tree:

-- Weights require exactly 2N*L*4 bytes = (2 * 989,109) * 21,946 * 4 bytes = 173.65 GB.

-- The memory of frequencies can be estimated as: 2N*a*g*4 bytes, being 'g' dependent on the number of gaps in the sequence. Since 'g' is not a constant value and changes in each node as the topology of the tree evolves, an exact value for the memory space used cannot be provided. In any case, most of the difference between the total memory used by VFT4 (272 GB) and the memory required only by the weights of the profiles (173.65 GB) is due to the frequencies.

Now we have included the above information in the revised manuscript (page 7).

[1] Price MN, Dehal PS, Arkin AP. FastTree: computing large minimum evolution trees with profiles instead of a distance matrix. Molecular biology and evolution 2009;26(7):1641–1650.

###################
###################

- Could you provide more insight into memory usage and program flow, particularly in relation to figures 4a and 4b? The plots indicate that the program typically maintains a modest memory footprint, averaging around 15-20 GB for the majority of its runtime. However, there is a notable spike in memory usage to approximately 80 GB at certain points. What is the reason behind this? How do you store intermediate results (conditional probabilities)?

****RESPONSE*****

The spike in the memory consumption is caused by the computation and storage of the profiles at each node of the tree. This information is now included in the revised text (page 8). The conditional probabilities, as we mentioned previously, were calculated using a profile (a C struct in the implementation). Each profile includes a frequency vector for each position, and the weighted average of its children’s profiles.

###################
###################

- The manuscript introduces several concepts such as level 3 and level 4 parallelization, as well as terms like "partitioning tendency window" and the utilization of a "tree partition algorithm" to accelerate spr movements. However, these concepts are mentioned without adequate explanation. It would greatly enhance the clarity of the manuscript to provide detailed explanations of these concepts, preferably accompanied by a figure for visual aid.

****RESPONSE*****

All these concepts have been clearly explained in the new revised "Parallelization strategy" section (pages 2, 3 and 4). As suggested, we have incorporated two new figures and a pseudocode detailing the tree partitioning algorithm to enhance clarity. Furthermore, we have provided clarification on the distinctions between different thread levels in the "New thread levels" section on page 4.
