# Supplementary material for: Efficient phylogenetic tree inference for massive taxonomic datasets: harnessing the power of a server to analyze 1 million taxa
Source: Gigascience. 2024 Aug 8;13:giae055. doi: 10.1093/gigascience/giae055 (PMC11308190; doi:10.1093/gigascience/giae055)
Supplement: giae055_Response_to_Reviewer_Comments_Revision_1 [file giae055_response_to_reviewer_comments_revision_1.pdf]

Author's Response To Reviewer Comments

We would like to thank the reviewers and editors for their insightful comments and suggestions about the paper. All the changes in the revised manuscript are highlighted in red. Detailed responses to reviewers are given below.

——

Review #3: I like the authors' new edits to the manuscripts. I believe the manuscript is now more readable, and the method is clearly explained. I only have a couple minor comments and suggestions that I hope will further improve the clarity of some sections:

###################
###################

- I may have missed it, but I did not see an explanation of what happens during the "tree partitioning" step when you refer to "a node is processed" or "computation of a node". Once the tree is split into non-overlapping subtrees, does each thread perform SPRs/NNIs and calculate likelihoods on its partial tree? It would be helpful to add a sentence about the type of work being done in the "Parallelization strategy" paragraph without changing anything in the "tree partitioning" section.

****RESPONSE*****

The explanation of the tree partitioning method is agnostic with respect to the specific operations performed on the nodes of the tree. This is why expressions such as 'computation of a node' are used. As stated in the paper (last paragraph, page 2), the most time-consuming routines for calculating the tree topology use the tree partitioning parallelization strategy, such as Nearest-Neighbor Interchanges (NNIs) and Subtree Pruning and Regrafting moves (SPRs). Similarly, the specific routines that use the parallel breadth-first traversal strategy are enumerated on page 4.

###################
###################

- In the same "tree partitioning" section, the concept of "penalty" is hard to follow. The penalty value determines whether a node can be processed in parallel: that is, if the size of the path (in nodes) from the considered node to the (partitioned) subtree's root for the current thread is larger than the penalty. If I understand correctly, in Fig. 1 penalty=2, and that is why node N is blue in t1 (the path from the root of subtree 1 to N is 3 nodes) while the parent of the two greens in t2 is red because the size of the path to the root of subtree 2 is 2 nodes. Perhaps you could include the above comparison of nodes N (t1) and the red parent of greens (t2) in your example for Fig 1. Additionally, the following sentence might be too complex and could be simplified: "which will be defined as the number of levels from subtree's root node that are excluded to ensure that all nodes have their dependencies satisfied". I think the word 'levels' is a synonym for nodes (on the path to the root), hence perhaps you could simplify the sentence using 'nodes' instead of introducing new terminology.

****RESPONSE*****

Following the suggestion of the reviewer, in order to clarify the explanation of the 'penalty value', we have rewritten the corresponding paragraph (page 2): "This dependency can be quantified as the distance between a node and the farthest one required for computation. We refer to this distance as the penalty value. This means that nodes located at levels lower than the penalty value from the subtree's root cannot be processed in parallel, as they have dependencies on nodes outside the subtree. In the example of Figure 1, the penalty is two, so P cannot be computed since it depends on the root (its grandparent), which lies outside the subtree. On the other hand, C, N, and all their children have their grandparents within the

subtree, allowing them to be computed without conflicts." On the other hand, we have used the standard definition of 'level of a tree' in graph theory: the number of edges between the root of the tree and a given node. This means that the level of the root node is 0, while its children are at level 1, and so on. In our discussion, we apply the same concept to the subtrees. We used 'levels' instead of 'nodes' since all nodes at a particular level of a subtree are treated equally.

###################
###################

- "partitioning method" section - Definition of "node weight". Perhaps "subtree weight" would be a better term since it clearly refers to a subtree of the tree, and the subtree is identified by its root node (in your definition). But I leave it to the authors to decide. \\

****RESPONSE*****

We kept the same notation. Note that this was explicitly explained in the text (page 3): "This way, the workload associated to process a subtree is the weight of its root node."
