# Supplementary material for: Efficient phylogenetic tree inference for massive taxonomic datasets: harnessing the power of a server to analyze 1 million taxa
Source: Gigascience. 2024 Aug 8;13:giae055. doi: 10.1093/gigascience/giae055 (PMC11308190; doi:10.1093/gigascience/giae055)
Supplement: giae055_Response_to_Reviewer_Comments_Revision_2 [file giae055_response_to_reviewer_comments_revision_2.pdf]

Author's Response To Reviewer Comments

We would like to thank the reviewers and editors for their insightful comments and suggestions about the paper.
