# Supplementary material for: Efficient phylogenetic tree inference for massive taxonomic datasets: harnessing the power of a server to analyze 1 million taxa
Source: Gigascience. 2024 Aug 8;13:giae055. doi: 10.1093/gigascience/giae055 (PMC11308190; doi:10.1093/gigascience/giae055)
Supplement: giae055_Reviewer_1_Report_Original_Submission — Michael Hiller -- 11/17/2023 Reviewed [file giae055_reviewer_1_report_original_submission.pdf]

Reviewer Report

Title: Efficient phylogenetic tree inference for massive taxonomic datasets: harnessing the power of a server to analyze one million taxa

Version: Original Submission **Date:** 11/17/2023

Reviewer name: Michael Hiller

Reviewer Comments to Author:

This paper presents VeryFastTree version 4, an update of the VeryFastTree method published initially in 2020.The new version features an advanced parallelization scheme that results in utilizing the maximum number of threads over nearly the full runtime of the program. Together with memory and other technical improvements, this makes VFT4 the fastest ML tree builder available, as the authors demonstrate in benchmarks. VFT4 also handles compressed file formats and features a disk computing functionality that stores data on the disk instead of RAM. For users with limited RAM machines, the latter feature enables them to compute trees, which would otherwise not be possible without upgrading RAM.The paper is well written and I only have minor suggestions:1) The abstract is very short and does not mention the new features build in VFT4. The features could be briefly mentioned (for those readers that don't read the full text).2) It would be helpful to add a figure that illustrates the new parallelization scheme.For example, the penalty parameter and parent / uncle swap is difficult to understand without seeing an illustration, especially because the penalty parameter is important for the new tree partitioning method.3) Why is level 3 and not level 4 the default level? Level 4 is faster and the authors mention that level 4 produces slightly higher accuracy. Is there a downside to level 4 parallelization?4) VFT4 is non-deterministic, which is great. My understanding is that the repeated runs of VFT4 with the same parameters result in identical results. However, how can users tell VFT4 to introduce variation in the tree space search? Is there something like a seed parameter that initializes a random number generator? If so, the user could test if different seeds result in similar or identical trees.

Level of Interest

Please indicate how interesting you found the manuscript: Choose an item.

Quality of Written English

Please indicate the quality of language in the manuscript: Choose an item.

Declaration of Competing Interests

Please complete a declaration of competing interests, considering the following questions:

- Have you in the past five years received reimbursements, fees, funding, or salary from an organisation that may in any way gain or lose financially from the publication of this manuscript, either now or in the future?
- Do you hold any stocks or shares in an organisation that may in any way gain or lose financially from the publication of this manuscript, either now or in the future?
- Do you hold or are you currently applying for any patents relating to the content of the manuscript?
- Have you received reimbursements, fees, funding, or salary from an organization that holds or has applied for patents relating to the content of the manuscript?
- Do you have any other financial competing interests?
- Do you have any non-financial competing interests in relation to this paper?

If you can answer no to all of the above, write 'I declare that I have no competing interests' below. If your reply is yes to any, please give details below.

none

I agree to the open peer review policy of the journal. I understand that my name will be included on my report to the authors and, if the manuscript is accepted for publication, my named report including any attachments I upload will be posted on the website along with the authors' responses. I agree for my report to be made available under an Open Access Creative Commons CC-BY license (http://creativecommons.org/licenses/by/4.0/). I understand that any comments which I do not wish to be included in my named report can be included as confidential comments to the editors, which will not be published.

Choose an item.

To further support our reviewers, we have joined with Publons, where you can gain additional credit to further highlight your hard work (see: https://publons.com/journal/530/gigascience). On publication of this paper, your review will be automatically added to Publons, you can then choose whether or not to claim your Publons credit. I understand this statement.

Yes Choose an item.
