# Supplementary material for: Efficient phylogenetic tree inference for massive taxonomic datasets: harnessing the power of a server to analyze 1 million taxa
Source: Gigascience. 2024 Aug 8;13:giae055. doi: 10.1093/gigascience/giae055 (PMC11308190; doi:10.1093/gigascience/giae055)
Supplement: giae055_Reviewer_2_Report_Original_Submission — Paschalia Kapli -- 2/26/2024 Reviewed [file giae055_reviewer_2_report_original_submission.pdf]

Reviewer Report

Title: Efficient phylogenetic tree inference for massive taxonomic datasets: harnessing the power of a server to analyze one million taxa

Version: Original Submission **Date:** 2/26/2024

Reviewer name: Paschalia Kapli

Reviewer Comments to Author:

The paper presents VFT4 - an upgrade version of the authors' own tool VFT3. The new updates as described in the paper include the application of SPR for exploring the tree space as well asthe support of new file formats (nexus and compressed files).The authors mainly compare the performance of VFT4 with VFT3 and FT2. My main concern with this manuscript is that there is insufficient detail on the new features of VFT4, often times paragraphs read too vague to understand. This of course does not mean the work should not be published; on the contrary I believe improvements to phylogenetic inference are important but the paper requires a substantial revision to explain its functionality, the concepts involved as well as experiments to demonstrate/evaluate its accuracy.Please find below a couple more specific comments:* The manuscript does not include any information regarding the scoring function used for ranking phylogenies. Does the tree search aim to find the maximum likelihood estimates of the parameters and the MLE tree, or is the search based on parsimony (e.g. the number of changes in the tree) or a combination of both approaches? The authors should clarify this. Also, based on some comments on "parent" and "uncle" nodes, it's unclear whether the program operates on rooted or unrooted trees. While recognizing that such information might have been described in prior publications, it's important for each manuscript to stand independently in this regard.* The manuscript needs a thorough discussion on how the correctness of results is assessed. Have simulation studies been conducted to evaluate the performance of the algorithm? Are the results compared against known phylogenies or validated against other phylogenetic software. If such comparisons are made, please specifying the software packages and datasets used for validation.To establish the reliability of VFT4, it would be advantageous to perform simulation studies using a set of datasets generated from real data. This approach would allow for the assessment of how frequently the program converges to the true phylogeny . Furthermore, conducting empirical analyses and comparing the results obtained from VFT4 with established software packages such as RAxML or IQ-TREE would instill confidence in the program's accuracy and robustness.* I find it hard to believe that single precision is beneficial for trees of that size (even for the Large dataset). Trees of such height will inevitably suffer from underflow when calculating conditional probabilities. How do you account for that? Even if some sort of numerical scaling is employed (e.g. adding a constant when reaching a threshold) I assume it is happening so often that it negates any benefit over using double precision (with scaling). Some discussion on this would be very useful.* "New parallel regions"The discussion on parallelization of tree traversals doesn't sound right. The overhead involved with traversing a tree structure, even with millions of nodes, is negligible compared to evaluating the scoring function (likelihood/parsimony/etc). My understanding is that a tree traversal involves generating an ordering for the tree nodes (e.g. postorder). It appears the authors may

be referring to parallelizing the entire process of topology and evaluation, but this requires clarification. Moreover, the purpose of tree partitioning and the utilization of breadth-first traversal in this context lacks explanation. The text suggests that the tree is divided into disjoint graphs so that threads can work independently (although this "work" is not explained), yet it still happens that threads may need to operate outside of their partitioned graph, and a penalty parameter is used to avoid this.Furthermore, it's unclear how topological changes (if that's what the authors describe) and branch lengths optimizations are executed in parallel on independent subtrees. Particularly, if the likelihood function is used for scoring, it is expected that changes necessitate re-evaluation of the likelihood by updating the conditional probabilities along the path to the root, assuming the remaining part of the tree is fixed (which I understand is not the case if you are working on differents parts in parallel). This is not described in the manuscript.* Finally, the section ends with the statement "On the other hand, in the parallel traverse approach, nodes at the same height level are computed simultaneously", which lacks clarity regarding what is computed and the methodology employed. The remaining part of the paragraph contains several undefined terms, such as "initial profiles" further exacerbating the confusion.To enhance clarity, I advice to replace this section with a more intuitive explanation of the parallelization strategy. This revised section should delineate what aspects of the program were parallelized, the previous state of the program and the benefits accrued from parallelization. A figure illustrating the parallelization would greatly enhance the presentation of the manuscript.Technical intricacies can be relegated to supplementary material for readers seeking detailed understanding.* Non-deeterministic deprecationThis section lacks informative content and delves excessively into details without contextualization. To improve clarity, replace the sentence 'certain sections of the code perform synchronized simultaneous modifications using mutex (a mechanism for controlling access to shared resources)' with 'critical regions', a standard term in the context of parallelizations. If the reader is not familiar with such a term, they will certainly not understand concepts like 'mutex' either. Additionally, the paragraph discusses some critical region, which had a non-deterministic behaviour, became deterministic, then it evolved back to non-deterministic again, and now, in VFT4, it's changed again to determinstic, without describing its function, the nature of deterministic and non-deterministic actions within it, or the implications for speed and memory footprint. I don't think this paragraph provides any meaningful information to the reader (at least not to me), and perhaps consider either clarifying or deleting it.* Some principles appear ot be misused, particularly regarding memory categorization. On page 3, the authors classify program memory (RAM) into static, dynamic and computation categories. However, the term "static memory" traditionally refers to memory that is allocated at compile time, and which remains unchanged throughout program execution. It appears that all three memory types mentioned by the authors are part of dynamic memory, allocated during the program execution. The description suggests that some dynamically allocated memory is transferred to disk when not actively utilized, a process which is akin to swapping managed by the operating system. The rationale behind implementing manual memory swapping within the program raises questions, especially considering that modern operating systems already employ sophisticated swapping mechanisms. Leveraging OS-managed swapping could obviate the need for custom memory management within the program, and thus the obvious question is why not just let the OS do theswapping when necessary?Moreover, the manuscript lacks clarity regarding the specific contents of memory being swapped to disk. While the authors mention static and dynamic memory is subjected to swapping (in my terminology, any kind of

memory allocated by the program at run-time), it remains unclear whether that encompasses things like intermediate likelihood/parsimony results at each node, which should be accessible by each thread (i.e. shared memory) and form the main bulk of memory scheme.Finally, I would imagine that the part of memory described as "computation" should be tiny compared to intermediate likelihood scores. Or do you refer to local NUMA memory as "computation"? In any case, describing the precise memory contents slated for swapping would be greatly appreciated by other software developers in the field.* Performance evalution: please explain what the -spr 4 -gamma -gtr switches mean. I assume a GTR+Gamma model is used with a discretized gamma approximation for rate heterogeneity using 4 categories, but this should be stated explicitly. Also, explain what the -spr 4 switch does.* Figure 1 and Figure 2 do not seem to match the text. I think the third cluster of bars (Ultra-large dataset) should be swapped between the figures?* Figure 3. Assuming VFT4 involves ML estimation using GTR-G4 and minimal site compression due to the amount of sequences, the memory requirement for the Ultra-Large dataset would be roughly ~ 1.4 TB [inner nodes * sites * states * rates * sizeof(float) = (989109-2)*21946*4*4*4] and that's accounting for only the conditional probabilities at inner nodes. Similarly, figs 4ab shows memory usage for the "Large" dataset using double precision. Based on my calculations the expected memory required for storing the conditional probabilities should be (274401-2)*1287*20*4*8 ~= 230 GB (assuming no site compression), yet the plotted max memory usage at a time is at most ~ 80 GB with 75% of time being at ~ 10GB. This is a huge discrepancy and should be explained/discussed whether VFT4 is doing something very smart or if there is a huge amount of gappy or identical sites affecting memory use.- Could you provide more insight into memory usage and program flow, particularly in relation to figures 4a and 4b? The plots indicate that the program typically maintains a modest memory footprint, averaging around 15-20 GB for the majority of its runtime. However, there is a notable spike in memory usage to approximately 80 GB at certain points. What is the reason behind this? How do you store intermediate results (conditional probabilities)?- The manuscript introduces several concepts such as level 3 and level 4 parallelization, as well as terms like "partitioning tendency window" and the utilization of a "tree partition algorithm" to accelerate spr movements. However, these concepts are mentioned without adequate explanation. It would greatly enhance the clarity of the manuscript to provide detailed explanations of these concepts, preferably accompanied by a figure for visual aid.

Level of Interest

Please indicate how interesting you found the manuscript: Choose an item.

Quality of Written English

Please indicate the quality of language in the manuscript: Choose an item.

Declaration of Competing Interests

Please complete a declaration of competing interests, considering the following questions:

- Have you in the past five years received reimbursements, fees, funding, or salary from an organisation that may in any way gain or lose financially from the publication of this manuscript, either now or in the future?
- Do you hold any stocks or shares in an organisation that may in any way gain or lose financially from the publication of this manuscript, either now or in the future?
- Do you hold or are you currently applying for any patents relating to the content of the manuscript?
- Have you received reimbursements, fees, funding, or salary from an organization that holds or has applied for patents relating to the content of the manuscript?
- Do you have any other financial competing interests?
- Do you have any non-financial competing interests in relation to this paper?

If you can answer no to all of the above, write 'I declare that I have no competing interests' below. If your reply is yes to any, please give details below.

I declare that I have no competing interests

I agree to the open peer review policy of the journal. I understand that my name will be included on my report to the authors and, if the manuscript is accepted for publication, my named report including any attachments I upload will be posted on the website along with the authors' responses. I agree for my report to be made available under an Open Access Creative Commons CC-BY license (http://creativecommons.org/licenses/by/4.0/). I understand that any comments which I do not wish to be included in my named report can be included as confidential comments to the editors, which will not be published.

Choose an item.

To further support our reviewers, we have joined with Publons, where you can gain additional credit to further highlight your hard work (see: https://publons.com/journal/530/gigascience). On publication of this paper, your review will be automatically added to Publons, you can then choose whether or not to claim your Publons credit. I understand this statement.

Yes Choose an item.
