# Supplementary material for: Efficient phylogenetic tree inference for massive taxonomic datasets: harnessing the power of a server to analyze 1 million taxa
Source: Gigascience. 2024 Aug 8;13:giae055. doi: 10.1093/gigascience/giae055 (PMC11308190; doi:10.1093/gigascience/giae055)
Supplement: giae055_Reviewer_3_Report_Revision_1 — Tomas Flouri -- 5/21/2024 Reviewed [file giae055_reviewer_3_report_revision_1.pdf]

Reviewer Report

Title: Efficient phylogenetic tree inference for massive taxonomic datasets: harnessing the power of a server to analyze one million taxa

Version: Revision 1 **Date:** 5/21/2024

Reviewer name: Tomas Flouri

Reviewer Comments to Author:

I like the authors' new edits to the manuscripts. I believe the manuscript is now more readable, and the method is clearly explained. I only have a couple minor comments and suggestions that I hope will further improve the clarity of some sections:- I may have missed it, but I did not see an explanation of what happens during the "tree partitioning" step when you refer to "a node is processed" or "computation of a node". Once the tree is split into non-overlapping subtrees, does each thread perform SPRs/NNIs and calculate likelihoods on its partial tree? It would be helpful to add a sentence about the type of work being done in the "Parallelization strategy" paragraph without changing anything in the "tree partitioning" section.- In the same "tree partitioning" section, the concept of "penalty" is hard to follow. The penalty value determines whether a node can be processed in parallel: that is, if the size of the path (in nodes) from the considered node to the (partitioned) subtree's root for the current thread is larger than the penalty. If I understand correctly, in Fig. 1 penalty=2, and that is why node N is blue in t1 (the path from the root of subtree 1 to N is 3 nodes) while the parent of the two greens in t2 is red because the size of the path to the root of subtree 2 is 2 nodes.Perhaps you could include the above comparison of nodes N (t1) and the red parent of greens (t2) in your example for Fig 1. Additionally, the following sentence might be too complex and could be simplified: "which will be defined as the number of levels from subtree's root node that are excluded to ensure that all nodes have their dependencies satisfied". I think the word 'levels' is a synonym for nodes (on the path to the root), hence perhaps you could simplify the sentence using 'nodes' instead of introducing new terminology.- "partitioning method" section - Definition of "node weight". Perhaps "subtree weight" would be a better term since it clearly refers to a subtree of the tree, and the subtree is identified by its root node (in your definition). But I leave it to the authors to decide.

Level of Interest

Please indicate how interesting you found the manuscript: Choose an item.

Quality of Written English

Please indicate the quality of language in the manuscript: Choose an item.

Declaration of Competing Interests

Please complete a declaration of competing interests, considering the following questions:

- Have you in the past five years received reimbursements, fees, funding, or salary from an organisation that may in any way gain or lose financially from the publication of this manuscript, either now or in the future?
- Do you hold any stocks or shares in an organisation that may in any way gain or lose financially from the publication of this manuscript, either now or in the future?
- Do you hold or are you currently applying for any patents relating to the content of the manuscript?
- Have you received reimbursements, fees, funding, or salary from an organization that holds or has applied for patents relating to the content of the manuscript?
- Do you have any other financial competing interests?
- Do you have any non-financial competing interests in relation to this paper?

If you can answer no to all of the above, write 'I declare that I have no competing interests' below. If your reply is yes to any, please give details below.

I declare that I have no competing interests

I agree to the open peer review policy of the journal. I understand that my name will be included on my report to the authors and, if the manuscript is accepted for publication, my named report including any attachments I upload will be posted on the website along with the authors' responses. I agree for my report to be made available under an Open Access Creative Commons CC-BY license (http://creativecommons.org/licenses/by/4.0/). I understand that any comments which I do not wish to be included in my named report can be included as confidential comments to the editors, which will not be published.

Choose an item.

To further support our reviewers, we have joined with Publons, where you can gain additional credit to further highlight your hard work (see: https://publons.com/journal/530/gigascience). On publication of this paper, your review will be automatically added to Publons, you can then choose whether or not to claim your Publons credit. I understand this statement.

Yes Choose an item.
